# Increased Wildfire Risk Driven by Climate and Development Interactions in the Bolivian Chiquitania, Southern Amazonia

**DOI:** 10.1371/journal.pone.0161323

**Published:** 2016-09-15

**Authors:** Tahia Devisscher, Liana O. Anderson, Luiz E. O. C. Aragão, Luis Galván, Yadvinder Malhi

**Affiliations:** 1 Environmental Change Institute‬, School of Geography and the Environment, University of Oxford, Oxford, United Kingdom; 2 Stockholm Environment Institute, Oxford, United Kingdom; 3 National Center for Monitoring and Early Warning of Natural Disasters, São José dos Campos, Brazil; 4 Remote Sensing Division, National Institute for Space Research, São José dos Campos, Brazil; 5 College of Life and Environmental Sciences, Geography, University of Exeter, Exeter, United Kingdom; 6 Posgrado en Geografía, Instituto de Geografía, Universidad Nacional Autónoma de México, Ciudad de México, Distrito Federal, México; Universidad de Chile, CHILE

## Abstract

Wildfires are becoming increasingly dominant in tropical landscapes due to reinforcing feedbacks between land cover change and more severe dry conditions. This study focused on the Bolivian Chiquitania, a region located at the southern edge of Amazonia. The extensive, unique and well-conserved tropical dry forest in this region is susceptible to wildfires due to a marked seasonality. We used a novel approach to assess fire risk at the regional level driven by different development trajectories interacting with changing climatic conditions. Possible future risk scenarios were simulated using maximum entropy modelling with presence-only data, combining land cover, anthropogenic and climatic variables. We found that important determinants of fire risk in the region are distance to roads, recent deforestation and density of human settlements. Severely dry conditions alone increased the area of high fire risk by 69%, affecting all categories of land use and land cover. Interactions between extreme dry conditions and rapid frontier expansion further increased fire risk, resulting in potential biomass loss of 2.44±0.8 Tg in high risk area, about 1.8 times higher than the estimates for the 2010 drought. These interactions showed particularly high fire risk in land used for ‘extensive cattle ranching’, ‘agro-silvopastoral use’ and ‘intensive cattle ranching and agriculture’. These findings have serious implications for subsistence activities and the economy in the Chiquitania, which greatly depend on the forestry, agriculture and livestock sectors. Results are particularly concerning if considering the current development policies promoting frontier expansion. Departmental protected areas inhibited wildfires when strategically established in areas of high risk, even under drought conditions. However, further research is needed to assess their effectiveness accounting for more specific contextual factors. This novel and simple modelling approach can inform fire and land management decisions in the Chiquitania and other tropical forest landscapes to better anticipate and manage large wildfires in the future.

## Introduction

Wildfires in Amazonia are expected to increase as the region is exposed to higher temperatures and water stress over the 21^st^ century [1–4]. Amazonian droughts such as that in the 1997/98 have been strongly related to El Niño events [5], and more recently to tropical Atlantic Sea Surface Temperature (SST) anomalies which have been linked to so called ‘mega-fires’ in the severely dry years of 2005 and 2010 [1,6,7]. A reduction in rainfall over Amazonia acts synergistically with other drivers such as land cover change, creating positive feedbacks that increase the susceptibility of the region to wildfires [8–13].

An increase in wildfire poses a threat to Amazon forests, affecting their structure and composition with the likelihood of a forest transition or dieback [14–19]. This in turn has effects on the global carbon balance further contributing to global warming [20,3]. Alencar *et al*. [20] estimated that widespread understory fires during the 1997/98 El Niño event resulted in 24–165 Tg of carbon committed emissions from the Brazilian Amazon through mortality, decomposition or combustion during subsequent fires. During the 2010 drought, Anderson *et al*. [21] estimated that old growth forest fires in the Brazilian Legal Amazon contributed 11.75–17.87 Tg of carbon to the atmosphere. During severe droughts, forest fires and tree mortality are likely to play a large contribution to carbon emissions from the Amazonia, potentially reversing its current net carbon sink [4]. Wildfires also have implications for human health, livelihoods of local populations and economies of Amazon countries [22–26].

Fire activity in Amazonia has predominantly occurred in and close to deforested areas [27,20,11,28]. This is because current wildfire in the region is almost entirely driven by human activity. Fire is widely used for the initial conversion of natural vegetation into agricultural and pasture fields (‘conversion fire’), and repeated burning has been used for the subsequent maintenance of deforested areas (‘maintenance fire’), such as pasture renewal and maintenance [29,11,30]. Shifts in the frequency, intensity and pattern of forest fires in Amazonia are closely linked to the agricultural frontier and represent a shift in the fire regime compared to historical patterns [31].

Recognising the anthropogenic and biophysical drivers of wildfire occurrence emphasizes the need to study the fire-climate-society nexus to anticipate and manage future wildfire risk. Future wildfire regimes will be a product of climate, land cover and land use change, and human management practices, all of which must be factored in. Modelling wildfire risk can be a useful method to better understand these interactions and can help not only to predict future wildfire impacts on the Amazon biome, but also to improve the design of climate change mitigation and adaptation strategies [3].

Within the fire research and practice community, fire risk refers to the probability of ignition both man- and lightning-caused [32]. In this study, we adopted this definition, but we also included an assessment of potential impacts linked to this probability of fire occurrence, which is important for anticipation and adaptation planning. Because the remotely sensed data used for modelling risk in this study–active fire detected by satellite sensors–do not distinguish between fire for agriculture (i.e. ‘conversion fire’ or ‘maintenance fire’) and wildfire (e.g. forest fire), we do not refer to probability of wildfire risk but instead to probability of ‘fire risk’, which includes a mixture of forest and non-forest agricultural, accidental and natural fires.

Some studies have modelled future fire risk in Amazonia considering not only drier weather conditions, but also different development pathways that can result in distinct deforestation trajectories [33–36]. Fire risk models have been developed for specific areas in Amazonia at finer resolution and using presence-absence data such as mapped burn scars [34,37], or integrating fire behaviour and propagation processes into a process-based fire model [36]. Mapping high-resolution burn scars and obtaining data on fire behaviour for different fuel environments is highly time consuming, thus applying these models to larger areas represents a challenge. In fact, the absence of data and understanding of fire dynamics in the different types of fuel environments of Amazonia is one of the major difficulties to predict wildfire in the region [38]. Fine-scale versions of fire risk models in this context require multiple components on ignition and propagation and numerous parameters that need to be adapted and calibrated to the diverse characteristics of Amazon landscapes [36].

Instead, a simpler and perhaps more useful modelling approach for such a diverse and large landscape is to adopt probabilistic modelling [35]. This technique can generate insights on fire risk based on the interplay of different spatial patterns that represent the incomplete information we have on the biophysical, climatic and anthropogenic factors that constrain the distribution of fire occurrence. On this basis, Silvestrini *et al*. [35] have recently developed a probabilistic model that allowed covering the whole of Amazonia using available fire occurrence data (NOAA-12 hot pixels), yet the study applied presence-absence modelling.

In this paper we apply maximum entropy modelling to predict probability of fire occurrence based on presence-only datasets [39] since defining a true absence of fire with hot pixels can be challenging when monitoring fire, if not undesirable [40,41]. This method, which is commonly used for species distribution modelling [39,42,43,44], has been applied only in very recent studies to model fire risk [45,46,41], and not yet in the context of Amazonia. Some of these models have used only land cover and anthropogenic variables to generate fire probability maps [45]. Renard *et al*. [46] and Arnold *et al*. [41] combined a series of climatic variables with static topographic, vegetation cover or distance to roads variables, but did not consider change in land use and land cover in their modelling task to assess how this dynamic would influence fire risk if interacting with more extreme climatic conditions.

In this study we address this gap and aim to assess how changes in land cover, land use and other anthropogenic variables (triggered by different development policies) interact with changes in climate to anticipate potential fire risk at the landscape level. We do this by simulating alternative future scenarios based on past and current conditions determining fire occurrence. Rather than studying Brazilian Amazonia, which is the usual focus of fire research in the Neotropics [47], we focus exclusively on a region located at the southern edge of Amazonia, the Chiquitania of Bolivia. The following questions underpin our research:

1. What are the main spatial determinants of wildfire occurrence in the Chiquitania region?
2. How do changes in climate and development trajectories affect future wildfire risk and what could be the potential impacts?
3. What strategies could have an inhibitory effect on wildfire risk?

The Chiquitania region is a stimulating case study in the lowlands of Bolivia to model fire risk and its sensitivity to changing climatic conditions. On the one hand, the extensive and well conserved seasonally dry tropical forest biome, which is a key and unique feature of this region [48–50], is exposed to marked seasonality and hence is susceptible to changes in climate and fire regimes. On the other hand, this region is undergoing a rapid expansion of its agricultural frontier, which adds to the reinforcing feedbacks we would like to study.

Recent remote sensing studies using MODIS data have estimated that an accumulated total of 9.6 million ha burnt in Bolivia due to forest fires between 2000 and 2013 [51]. Most of these forest fires (71%) occurred in the Department of Santa Cruz where the Chiquitania region is located. During the 2010 drought that affected the region (S1 Fig) raging wildfires burned about 2 million ha of forests across the Department of Santa Cruz, leading to a national state of emergency [51].

Risk of wildfires may increase in the future as the Chiquitania region faces drier and more seasonally extreme climatic conditions, associated to different climate modes such as El Niño–Southern Oscillation, the Pacific Decadal Oscillation [18,52], and tropical Atlantic STT anomalies related to the Atlantic Multidecadal Oscillation [1,6]. Using a regional climate model (PRECIS ECHAM4 results under the SRES A2 high-end emissions scenario), Seiler [53] assessed that temperature in the Bolivian lowlands (i.e. areas below 500 mamsl) can be expected to increase by about 1.3°C by 2030 and 4.7°C by 2100. The projections also showed that seasonality might intensify, with increased rainfall during the rainy season (DJF months) and less precipitation during the dry season (JASO months). Based on global circulation models (CMIP5 RCP8.5), Seiler *et al*. [54] assessed a projected increase in temperature of 2.5–5.9⁰C for Bolivia by the period 2070–99. Most projections for the Bolivian lowlands showed significant decrease in annual accumulated rainfall, with less precipitation during the drier months from July to November, and significant change in inter-annual rainfall variability [54].

Changes in the region are also driven by policies fostering frontier expansion and immigration, both of which are spreading the use of fire. Since mid-2000s a wave of migration driven by post-neoliberalism policies is supporting the settlement of new farming communities [55]. This combines with recent national plans to expand the agricultural frontier and the road network in the region [56,57] based on socio-economic development priorities, food security and sovereignty (e.g. Law N337 [58], Law N650 [59]). In 2015, discussions between the national government and the agro-industry sector led to a national target to expand the agricultural frontier to 13 million ha by 2025 [60–62]. By 2010 about 4.6 million ha were deforested in the Bolivian lowlands [63], which means that the new national target would require expanding the frontier by almost 10 million ha in the next decade; an action that will undoubtedly further spread the use of fire into new forest frontiers.

## Study Area Description

**Fig 1:**
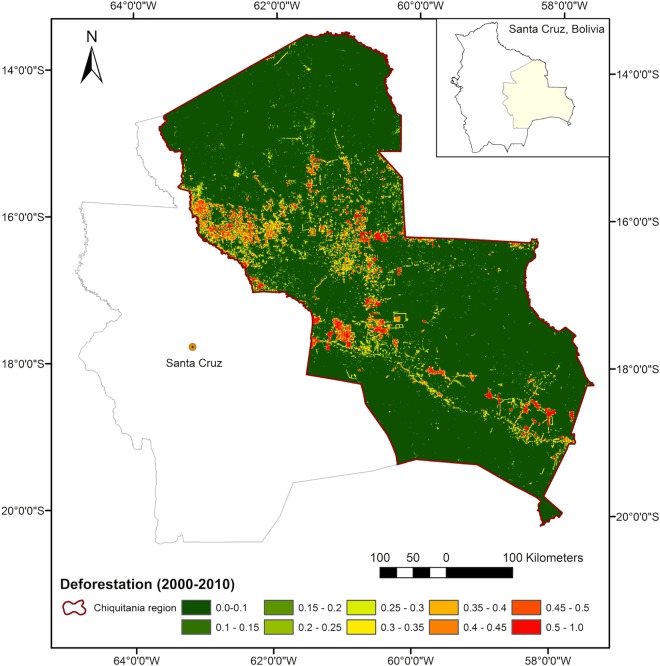
Our case study the Chiquitania region in the Department of Santa Cruz, Bolivia. Area is delimited by the boundaries of the Chiquitano Model Forest [69]. The map shows the deforestation pattern in the region from 2000 to 2010 according to data generated by FAN [70].

The Chiquitania region is located in the Department of Santa Cruz, Bolivia. This case study region is part of the Chiquitano dry forest ecoregion that spreads over Bolivia, Brazil and Paraguay linking the Amazon rainforests to the north with the Gran Chaco shrublands to the south [48]. To promote conservation and sustainable development in the Bolivian lowlands, the Chiquitania was recognised as a ‘Model Forest’ in 2005 [64,65] (Fig 1). The Chiquitano Model Forest (CMF) covers a bit more than 200 thousand km^2^ where the predominant natural vegetation is tropical dry forest with semi-deciduous trees, intertwined with grasslands and shrubbery of the woody savanna *cerrado* [66,48,49]. By 2010, about 54% of the Chiquitania was covered by seasonally dry tropical forest, and about 80% of the territory was used for cattle ranching including mixed agricultural use and forested rangeland [67]. Land tenure in the Chiquitania region is concentrated in the livestock sector, which together with the forestry sector contributes to about 90% of the regional economy [48].

The seasonally dry tropical forests in the Chiquitania grow on relatively fertile soils. Understory presence of C4 grasses in these closed canopy forests is infrequent and natural fire is rare [68,49,50]. On the contrary, fire in the grasslands of the savanna *cerrado* is more common. Because dry forests and grasslands occur as mosaics, it is necessary to consider their inter-connections at the landscape level [50].

The regional climate is characterised by a marked dry season from July through November. Mean annual precipitation in the central area of the region is 1129 mm varying between 500 and 1710 mm between years [66]. Based on NASA TRMM data for the region (2000–2013) an average of 6 months a year (starting in April/May) receive <100 mm, with the driest months being July and August (around 20±3 mm month^-1^). Temperature varies little throughout the year with daily means of 24–25°C. Northern winds are common throughout the year and oscillate between 3.7 and 18.5 km h^-1^. Although less frequent, southern winds during the dry season are more intense, dry and cold, and can affect the spread of fires.

There is a long history of fire use in the traditional production systems of the Chiquitania [71,72]. Wildfires are recognised as part of the disturbance regime of the region, however in recent years they have become more dominant and difficult to manage. Wildfires are mainly anthropogenic and closely related to human activities such as slash-and-burn agriculture, pasture management, waste burning, hunting, and others [72,30].

## Materials

### Modelling approach

We used maximum entropy (MaxEnt) modelling to predict fire risk and simulate future scenarios. MaxEnt is a general-purpose method that uses statistics and machine learning to make predictions or inferences from incomplete information, suitable for all existing applications involving presence-only datasets [39]. MaxEnt estimates a target probability distribution by finding the probability distribution of maximum entropy (i.e. that is most spread out) subjected to a set of constraints that represent our incomplete information about the target distribution. This information is contained in a set of spatial variables and functions thereof (i.e. environmental features) that characterise the environment of the study area and not only the conditions at presence sites. The sample points used as presence data in the model were localities where fire was observed (i.e. MODIS-detected hotspots). The model predicts suitability for fire occurrence as a function of the environmental features, where “the expected value of each feature should match its empirical average” ([39], p.234). In other words, the model output indicates the areas that satisfy the conditions for fire occurrence based on constraints defined by the environmental features informing the model. In this sense, this model builds on what is known, but carefully avoids anything that is unknown [39].

Our model resolution was 1 km to keep consistency with the 1 km^2^ MODIS footprint to detect fires used as sample points (presence data) in the model. This also helped to minimize potential geo-location biases of fires. It was also a relevant resolution given the geographic scale and grain needed for the modelling task, particularly as we combined climatic variables that are more suitable for meso-scale models with land cover and land use variables that have more effect at a micro-scale.

Maximum entropy modelling offers certain advantages to model fire risk over more traditional presence-absence modelling methods. With MaxEnt, background values in the environment where fire has not been observed are not treated as absences during the modelling task, instead used as constraints on the unknown probability distribution. This makes this method more appropriate for using active fire detection products such as MODIS hotspots where true absence is difficult to define (i.e. fires can occur that are undetected by MODIS). It also helps in cases where sample points are scarce and distributed over a large geographical area (not the case in this study). Moreover, the model output of relative probability of presence generated with MaxEnt is continuous, which allows making a finer distinction between the levels of fire risk in different areas. This is more useful to inform management decisions (See S1 File, also for some limitations of the method).

### Model variables

Different variables with temporal and spatial correspondence were processed to build a maximum entropy model of fire risk for the CMF region. Variables included environmental, socio-economic and climate-related features relevant to fire occurrence in the period 2000–2010. This period was selected because the data quality and availability were suitable for model input, while datasets for years prior to 2000 were not always complete. The period also captured important changes that have lately affected the region, which were necessary to consider in model development to simulate scenarios that assume these changes intensify in the future. S1 Table shows all the variables processed and tested individually and in different combinations to build models of fire risk. Table 1 lists the most significant variables selected to obtain more parsimonious models (Maps in S2 and S3 Figs). Dynamic variables that were manipulated in the scenario simulations are highlighted in Table 1 and include deforestation, roads, protected areas, and climate-related variables (See more detailed assumptions for changes in these variables in the section ‘Future scenarios’). Data for variables used as model inputs were obtained with permission of local research organisations signing Memoranda of Understanding. Other spatial data were publicly available and no specific permissions were required. All sources are included in the References and indicated in Table 1 and S1 Table.

**Table 1:** Selected variables for fire risk modelling.

Variable	Description	Original resolution§	Post-processing unit	Source
Chiquitano shrubland	Land cover category important for fire occurrence obtained from the 2010 land cover and use map of Bolivia	50 m	%	UTNIT [67]
Grassland	Land cover category important for fire occurrence obtained from the 2010 land cover and use map of Bolivia	50 m	%	UTNIT [67]
Deforestation#	Deforestation between 2000 and 2010 estimated from the map of deforestation to 2000, 2005 and 2010 for the lowlands of Bolivia	30 m	%	FAN [70]
Roads#	Euclidean distance to roads weighted by paved and unpaved roads to 2008	n/a	m	FCBC [69], ABC [76]
Population density	Kernel density of human settlements weighted by the population of each Municipality area within the CMF region	n/a	nw‡ km^-2^	FCBC [69], INE [77]
Protected areas#	Different categories of protected areas and indigenous land	n/a	cat	SERNAP [78], FCBC [69]
Temperature#	Annual anomalies of mean temperature (2000–2010 baseline) using monthly land surface temperature data from MODIS Terra MOD11C3	0.05°	kelvin†	NASA USGS [79]
Precipitation#	Annual anomalies of MCWD (2000–2010 baseline) based on monthly rainfall data from the Tropical Rainfall Measuring Mission	0.25°	mm	NASA TRMM [80]
Hotspots	MODIS Aqua and Terra MCD14ML high-confidence hotspots for the period 2001–2010 (version 5.1)	1 km	count km^-2^	NASA FIRMS [81]

MCWD: maximum climatological water deficit calculated applying a threshold of 100 mm to the dataset (See S2 File for details)

§ Post-processing resolution for all variables is 1 km

# Variables manipulated in future scenario simulations

‡ Number (n) of human settlements weighted (w) by population

† Multiplying by a scale factor of 0.02

Recent studies found that protected areas (PAs) can limit the spread of forest fires in other regions of the Amazonia [73,74,35]. Despite this variable had only minor contribution to overall model performance, we decided to include it in order to test if the establishment of new PAs in strategic locations would help inhibit fire risk. In Bolivia PAs have different designation and can be managed at different scales, from the national (PA) level to the departmental (DPA) and the municipal (MPA) levels, with associated implications for activities allowed within their boundaries, and resources and capacity to monitor the areas. Besides PAs, in the 1990s the Bolivian state also recognised indigenous land. Since 2011 these areas are referred to as *Territorio Indigena Originario Campesino* (TIOC) and currently most are consolidated with land title [56]. In the lowlands of Bolivia, the TIOCs occupy large areas and include primary forests in locations of less road connectivity. Because of this, they have an important role to play in forest conservation if managed sustainably [75]. For this reason, in this study we included both TIOCs and PAs.

We used MODIS-detected Aqua and Terra MCD14ML high-confidence (>80%) hotspots as sample points for the model. First we analysed hotspots for the period 2001–2013 (only Terra in 2001) to gain a broad understanding of the spatial and temporal distribution of fire occurrence in the region. We then calibrated the model using 2001–2010 hotspots for the region to ensure temporal correspondence with the environmental features.

MODIS can routinely detect both flaming and smouldering fires around 1 km^2^ in size. Under very good observing conditions even smaller flaming fires of about 50 m^2^ can be detected [82]. Hotspots are recorded when one or more fires (≥227°C) are identified within the 1 km^2^ footprint. Because active fire detection by MODIS does not distinguish between different fire types, MCD14ML hotspots provide a proxy for the occurrence of biomass burning events that may be associated to conversion fire, maintenance fire and wildfire [83].

We acknowledge that the MCD14ML hotspots present biases in active fire detection due to factors such as: fire that started and ended between satellite overpasses, fires that are too small or cool to be detected by the footprint, cloud cover, heavy smoke, or tree canopy that may completely obscure fires such as small understory fires [82,84]. However, we decided to use MODIS hotspots for this modelling task based on the following careful considerations. First, in other studies small-size burned scars have shown to have little contribution to overall burned area detected with MODIS [85,84]. Second, despite the Landsat multispectral data has proved suitable to map burn scars [86,31,84], the lower temporal resolution of Landsat combined with high cloud cover makes this sensor less ideal and more time intensive to map burn scars over large spatial and temporal scales.

Third, a validation implemented in Amazonia showed that only 13% of a 1 km^2^ MODIS hotspot needs to be occupied by an active fire to achieve high detection confidence, denoting the accuracy of the MODIS fire algorithm [87]. Fourth, the omission error by MODIS fire detection is less problematic when using MaxEnt because the model uses presence only data. This means that even with omission errors (e.g. missing small understory fires), the high confidence MCD14ML hotspots are appropriate to train the model because these locations–where we have more certainty that fire has occurred–will serve to identify other areas in the region with similar environmental characteristics suitable for fire occurrence. Commission errors, on the other hand, are more problematic because they could lead to false alarm with high fire risk in areas where fire has not occurred. This is indeed a limitation, however it is likely to be minimized as commission errors occur only under the following circumstances: (i) sensor saturation from a high-heat fire duplicating the hotspot in line, yet would have minimal influence on the result due to the spatial scale we covered; (ii) large temperature difference between land cover types (e.g. boundary of forest and bare soil), although these forests would be more susceptible to fire so it would not entail a conceptual error in terms of spatial location of the fire probability; and (iii) targets with high temperature (e.g. rocks, sandy soils), however these hot pixels are sparse and would have minimal influence on the results due to the spatial scale.

Fifth, the dataset we used presents significant advantages such as global coverage, high temporal resolution and time accuracy, which makes it a convenient, systematic, and reliable dataset to use for input in modelling that can be replicated elsewhere. Note we chose a long-running dataset that provides one systemic observation of fire patterns over large temporal and spatial scales and avoided using multiple datasets from different satellite sensors, which largely detect different fires and are not necessarily complementary [88]. Finally, it is also important to recognise that the government agencies in Bolivia are already using remotely sensed hotspots in their fire monitoring activities. This model could therefore complement their work, and be used to enhance their capacity to anticipate fire risk.

## Methods

### Data processing and selection

Each variable used for the fire risk model was processed and converted to 1 km resolution using a combination of different tools as described in detail in S2 File. First we ran the MaxEnt model with each variable on an individual basis to assess their importance. Then we applied a factorial approach where we grouped the variables into development, environment, and climate-related variables. Next, we tested all variables together, and at each run we removed the variables that were not contributing significantly to model gain following the principle of parsimony. For the analyses above we used the jackknife test, the response curves of each variable, and their percent contribution and permutation importance [89]. By the end of this process, we had selected significant variables and eliminated variables or predictors that were highly correlated and included information that was already contained in other variables (i.e. avoiding collinearity between predictors). Selected variables used in the more parsimonious fire risk model are listed in Table 1.

### Model testing, calibration and validation

Model goodness-of-fit was tested and compared using the Area Under Curve (AUC) score. The AUC score was calculated using the threshold-independent receiver operating characteristic (ROC) analysis adapted to presence-only modelling [39]. The AUC score reflects the ability of the model to distinguish presence from random background where an AUC of 0.5 means that the model does not better than random. In addition, for each model run (500 iterations), we set aside 25% of the sample records for testing (validation) using a random seed each time. The test AUC scores were also estimated to compare. Model outputs that showed higher false alarm were penalised, i.e. preference was given to more conservative models on the premise that risk maps should encourage intervention only when there is true high probability of fire occurrence.

The best-performing model (referred to as ‘model 2010’ hereafter) with highest AUC score was calibrated with hotspots corresponding to the period 2001–2010, equivalent to 88,883 data points. This model was also calibrated using maximum climatological water deficit (MCWD, see S2 File) and temperature anomalies for the dry year 2010. We decided to calibrate the model to 2010 climatic conditions to encompass a higher range of variability, which was preferred so that we could subsequently run future scenario simulations considering extreme dry conditions (i.e. as an analogue to seasonality becoming more intense due to climate change). Other model settings included 0.5 default prevalence, and a regularization multiplier set to 1, which allowed reducing model over-fitting while avoiding to estimate a distribution that is so spread out that could lead to false alarm.

The ‘model 2010’ was then used to generate a projection for 2009 using the MCWD and temperature anomalies for 2009. We selected the year 2009 because it was considered a normal/wet year (see S1 Fig) and therefore an appropriate case to test the model performance in validation. Model validation using the 2009 projection was conducted estimating threshold-independent specificity [39]. For this we assessed the distribution of observed hotspots in the projected year, equivalent to 3,991 data points, falling in each probability threshold of the projection output generated by the model. An additional cross-validation was implemented with the 2009 projection. This entailed replicating the model run 10 times (500 iterations each) and randomly splitting the presence data into a number of equal-sized groups. Models were run leaving out each group in turn. Projection outputs were compared and the average output was used in the impact analysis explained in the following sections.

### Future scenarios

We built three future scenarios considering different possible development trajectories in the Chiquitania region, mainly based on national development and land use policies (Table 2, Maps in S4 Fig). Most development policies for the country envision concrete goals for 2025 [59]. Consequently we considered this time horizon to be politically relevant to generate scenarios that can inform decisions. Each 2025 scenario was run under the conditions of a normal/wet year (2009 MCWD and temperature anomalies) and under the conditions of a drought year (2010 MCWD and temperature anomalies) as analogy of what could happen under future drier conditions due to climate change. Scenario simulations were replicated 10 times (500 iterations each) and average outputs were used in subsequent analyses.

**Table 2:** Brief scenario descriptions and assumptions.

	Sustainable growth (Scenario A)	Business as usual (Scenario B)	Rapid growth (Scenario C)
**Assumptions and change in variables†**	• Implementation of new conservation policies. • More Municipal PAs and TIOCs, new Departmental PAs are established in areas of high fire risk with low land tenure by 2009. • Current road network is maintained. • Intensive systems are encouraged instead of rapid frontier expansion, deforestation rate decreases after 2013.	• The economic and deforestation trends of 2000–2010 continue. • The paved road network is moderately expanded. • More Municipal PAs and TIOCs, no new Departmental PAs are established. • Regional wildfire risk management strategies are not envisaged.	• Economic policies lead to the expansion of the agricultural frontier to reach national target of 13 million ha in 2025. • The paved road network is expanded according to national projections. • New protected areas and wildfire risk management are not envisaged.
**Assumption sources**	Deforestation: 2025 deforestation maps for the Bolivian lowlands under scenario A by Tejada *et al*. [62] (AmazAlert project). • PAs and TIOCs: SERNAP [78], FCBC [69], and authors’ assumption.	• Roads: 2020 projection by the ABC [76].Deforestation: 2025 deforestation maps for the Bolivian lowlands under scenario B by Tejada *et al*. [62] (AmazAlert project). • PAs and TIOCs: SERNAP [78], FCBC [69].	• Roads: 2020 projection by the ABC [76]. • Deforestation: 2025 deforestation maps for the Bolivian lowlands under scenario C by Tejada *et al*. [62] (AmazAlert project).

PAs: protected areas, TIOCs: indigenous land (*Territorio Indigena Originario Campesino)*, SERNAP: National Service of Protected Areas (*Servicio Nacional de Áreas Protegidas*), FCBC: *Fundación para la Conservación del Bosque Chiquitano*, ABC: Bolivian Road Administration (*Administradora Boliviana de Carreteras*).

† All other variables in the simulations were maintained unchanged. S4 Fig presents the maps showing the change in dynamic variables.

The **sustainability scenario A** assumed implementation of new conservation policies like the new Environmental and Mother Earth laws and plans for integrated forest and land management under the Joint Mechanism introduced by the Bolivian government in 2012 (Law N300 [90], Decree N1696 [91]). This scenario was the only one to consider a regional wildfire risk management strategy. Under this scenario, we assumed a slower socio-economic growth, and the establishment of additional PAs and TIOCs. We focused on the establishment of new DPAs in areas of high fire risk, because this category showed the smallest relationship with fire occurrence in the model (S5 Fig). We assumed that locating new DPAs in areas of high fire risk could be a potential measure for fire risk management, also given the increased monitoring activities in these areas. Finally, more intensive agriculture and livestock production systems are encouraged in this scenario instead of accelerating the expansion of the agricultural frontier.

The **business as usual scenario B** assumes current 2000–2010 developmental trends continue. Deforestation is led mainly by cattle ranching and mechanised agriculture, and also due to the immigration of new settlers in the region. The paved road network is expanded, although it does not fulfil national projections due to relatively moderate economic growth. Some new MPAs and TIOCs are established, but not DPAs.

Under the **rapid growth scenario C**, a series of economic policies implemented in the region accelerate the expansion of the agricultural frontier and boost the country’s socio-economic growth. The agreement between the national government and the production sectors is implemented, expanding the agricultural frontier to 13 million ha in the lowlands of Bolivia [62]. The paved road network is developed according to national projections. The economy grows fast but is not diversified, based mainly on extractive activities such as agriculture, livestock and mining even in protected areas. New protected areas and fire risk management are not envisaged.

### Analysis of potential impact

Potential impacts caused by fire were assessed in terms of forest biomass loss and socio-economic implications. This analysis was conducted for all scenarios. To compare potential impacts of different model outputs we focused on areas of high fire risk. We defined these areas as cells with probability values >0.5 in the fire risk maps, grouping the last two classes of risk suggested by Ferrarini [40] to interpret outputs generated with MaxEnt for fire modelling: 0–0.25 low risk, 0.25–0.5 moderate risk, 0.5–0.75 high risk, and 0.75–1 extreme risk. We acknowledge that this analysis may over-estimate area at risk because it assumes that the whole pixel is at risk to be burned, when in fact MODIS hotspots used in the model only provide information on location of fire occurrence and not area. However, given that MCD14ML is known to under-estimate fire occurrence (particularly understory fire) this might be compensated to some extent. Also, we note that recent studies [21] have shown there is good agreement between MODIS hotspots and burned area, demonstrating the potential capability of using active fire information to estimate burned area and biomass loss over large areas.

To estimate a potential envelop of biomass loss and consider uncertainty, we used aboveground biomass (AGB) datasets developed by three studies: (i) Yu *et al*. (*Unpublished data*) building on the Saatchi *et al*. [92] dataset, (ii) Baccini *et al*. [93], and (iii) Mitchard *et al*. [94]. The first two datasets are based on remote sensing data, while the last one is based on a geostatistical model of field data for Amazonia. By using multiple datasets we captured a systemic error in the AGB estimates, which is more conservative than the estimated error produced by the method employed in each study. With each dataset, mean AGB was calculated for each probability threshold of the fire risk maps generated with the model. An average AGB based on the three datasets was then calculated for each probability threshold. Loss of AGB due to fire was estimated using the following equation:

$$B_{l}=(1-\alpha )B_{i}$$ (1)

where *B*_*l*_ is the potential aboveground biomass loss (Mg ha^-1^) after fire, *B*_*i*_ is the initial aboveground biomass (Mg ha^-1^), and *α* is the proportion of AGB remaining post-fire ranging from 0.7084 [21] to 0.90 [95]. The range of potential AGB loss estimated with Eq (1) accounts for biomass loss between 1 and 5 years after fire occurrence, without accounting for fire recurrence. More details are provided in S3 File.

Finally, potential socio-economic implications of the fire risk maps were estimated using the current land use and land cover (LUC) map for Bolivia updated to 2010 [67], and the Land Use Plan (PLUS) map for the Department of Santa Cruz updated to 2009 and processed for the CMF region (S2 Table). Using zonal statistics, the mean probability of fire occurrence was estimated for (i) each category of the LUC map, to assess the differences between a wet and a dry year, and (ii) each category of the PLUS map, to assess the differences between the scenarios with and without the effects of climate change (i.e. severely dry conditions).

## Results

### Observed distribution of fires

**Fig 2:**
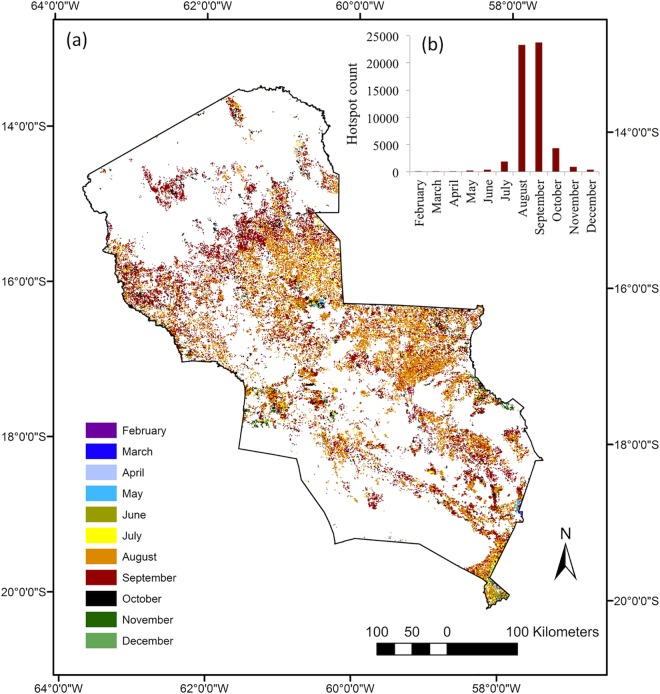
Peak months of hotspot occurrence in the Chiquitano Model Forest, Department of Santa Cruz, Bolivia. (a) MODIS MCD14ML high-confidence hotspots are coloured according to the month with the highest number of hotspots during the period 2001–2013. (b) Histogram showing total number of MCD14ML high-confidence hotspots per month in the period 2001–2013 for the Chiquitano Model Forest region. In the modelling task we excluded hotspots in 2011–2013 to maintain temporal correspondence with the environmental variables used in the model.

Fires usually occurred during the dry season due to a combination of favourable conditions such as dry biomass, weather and land management practices. The months of August and September were identified as peak fire months based on the total number of high-confidence hotspots counted per month during the period 2001–2013 (Fig 2). About 83% of fires occurred between these two months and 93% between August and October (Fig 2B), with considerable inter-annual variability. Although peak months are well distributed across the region, the northern part of the region showed a larger number of hotspots in September (Fig 2A). This indicated that this area, which is covered by more humid Amazon forests, required longer time to show the flammability conditions necessary for forest fire outbreaks to occur.

### Model performance

The development, environment, and climate-related variables had different importance for model gain. The factorial analysis showed that when the 2010 hotspots were excluded, the models with only development variables (i.e. land use and land cover, roads and deforestation) showed the highest AUC scores (0.706). In general, 2009 climate-related variables showed less contribution to model gain. When including 2010 hotspots in the model, 2010 climate-related variables became more important for model gain (AUC score 0.704). This indicated that climatic variables become more important drivers of fire occurrence in extremely dry years such as 2010.

The best-performing parsimonious model calibrated with 2001–2010 hotspots (i.e. ‘model 2010’) generated an AUC of 0.70. This is comparable to other fire risk models developed with MaxEnt, which obtained similar performance results with an AUC of 0.72 [45] and 0.88 [46]. The contributions of the variables included in the ‘model 2010’ were: Chiquitano shrubland (27.7%), road network weighted by paved and unpaved roads (22.7%), deforestation between 2000 and 2010 (17.1%), density of human settlements weighted by population in each Municipality (15.8%), mean temperature anomalies (6.7%), grasslands (4.6%), maximum climatological water deficit (MCWD) anomalies (3.3%), and protected areas and indigenous land (2.2%) (See jackknife test in S6 Fig). The protected areas variable was kept in the final model despite its low contribution to model gain because we wanted to assess the effect of establishing protected areas in strategic locations to inhibit future fire risk.

### Modelling fire risk in dry and wet years

**Fig 3:**
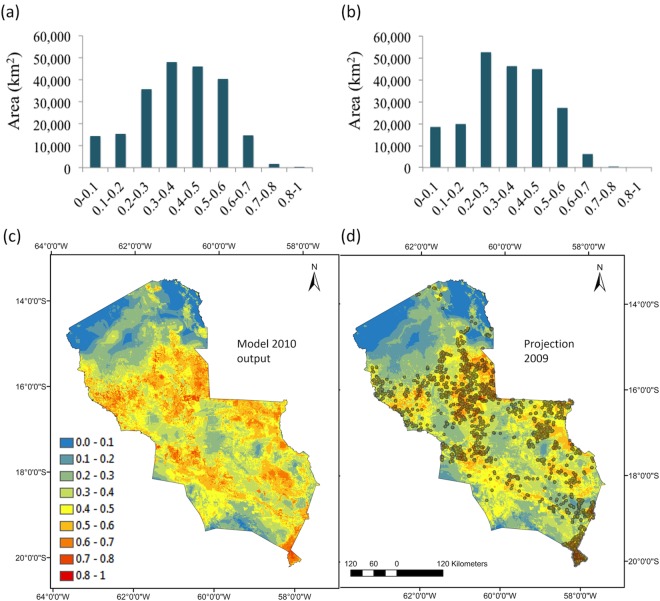
Histograms show the frequency distribution of 1 km resolution cells (area in km^2^) across the different probability thresholds of fire occurrence in (a) the model 2010 output using 2010 temperature and MCWD anomalies, (b) the 2009 projection of using 2009 temperature and MCWD anomalies. Maps show the fire risk based on the same probability threshold values for (c) the model 2010 output and (d) the 2009 projection overlaid by 2009 MCD14ML hotspots for validation.

The area with high fire risk (>0.5 probability) under dry climatic conditions (2010 MCWD and temperature anomalies) was 69% larger than in the projection output for the normal/wet year 2009. About 56,700 km^2^ were at high fire risk in the 2010 model compared to 33,500 km^2^ in the 2009 projection (Fig 3A and 3B). This was most likely driven by the difference in mean temperature anomalies between 2009 and 2010 (See S3 Fig). In a dry year, the model captured the higher risk of fire occurrence in the northern area of the region, which is covered by more humid Amazonian forests that are generally less prone to fire (Fig 3C). Validation of the 2009 projection conducted using 2009 MCD14ML hotspots (Fig 3D) showed high sensitivity with almost 60% of total observed hotspots that year falling above the 0.5 probability threshold (S7 Fig).

### Scenarios of fire risk for 2025

**Fig 4:**
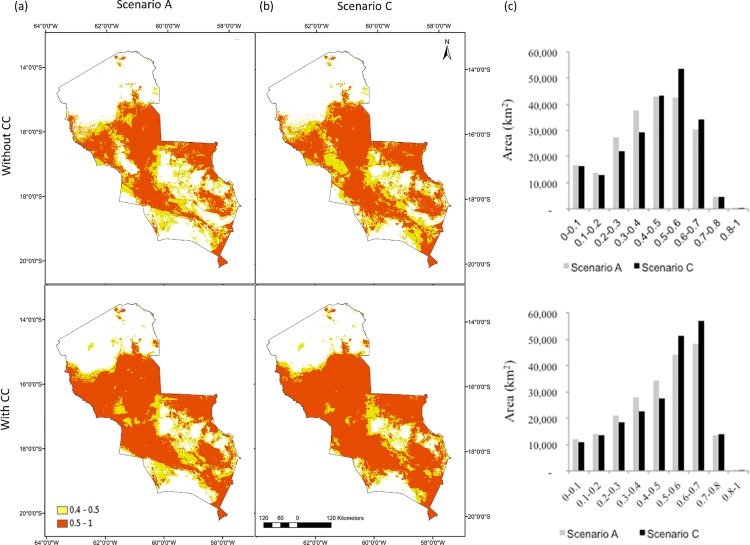
Simulations of fire risk using the model 2010 for (a) sustainability scenario A without climate change (CC) and with CC and (b) rapid growth scenario C without CC and with CC. To help visualisation, we coloured high fire risk area (>0.5 probability) red in the map. (c) Histograms show the frequency distribution of 1 km resolution cells (area in km^2^) across the different probability thresholds of fire risk in scenarios A and B without CC (top) and under drier climatic conditions (below).

Simulations for 2025 were based on different development trajectories in the Chiquitania. Comparing simulation results (Fig 4), the area of high fire risk (>0.5 probability) was 20% less in the sustainability scenario A (77,600 km^2^) than in the rapid growth scenario C (92,500 km^2^). Scenario A showed a partial decrease in fire risk where new DPAs were established (Fig 4A). Road network development and deforestation increased area at high fire risk (>0.5 probability) by 1.5 times in future scenario B and by 1.8 times in scenario C compared to the 2009 projection. A combination of land use change and dry climatic conditions increased the area at high fire risk by 1.2 times comparing scenario C (Fig 4B with CC, equivalent to about 122,800 km^2^) with a current dry year (model 2010 output) and by 2.6 times with a current wet year (2009 projection).

### Potential fire impacts on biomass

**Fig 5:**
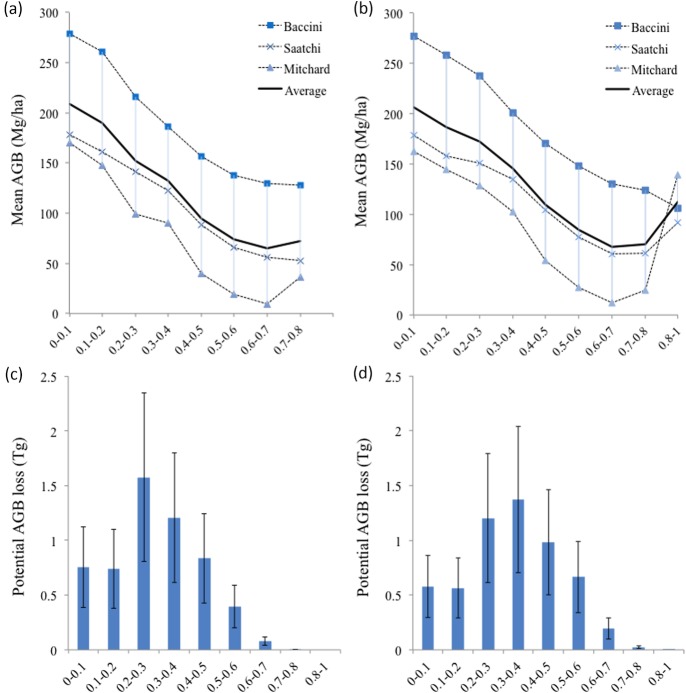
Curves show mean aboveground biomass estimated using three different datasets and their average for each probability threshold of fire risk for (a) the 2009 projection using 2009 temperature and MCWD anomalies and (b) the model 2010 output using 2010 temperature and MCWD anomalies. Bars show potential biomass loss for each probability threshold estimated averaging Yu et al. building on Saatchi et al. [92], Baccini et al. [93] and Mitchard et al. [94] datasets for the region and using Eq (1) for (c) the 2009 projection and (d) the model 2010 output. Error bars correspond to the range of AGB loss proportion considered in Eq (1).

Although the three datasets used to estimate potential AGB loss generated different estimates, they followed a similar pattern showing an envelope of uncertainty (Fig 5A and 5B) with mean loss factor 0.195 (±0.096) based on Eq (1). This pattern showed that in both wet and dry years the higher mean AGB values were in the lower risk probability ranges, and that in general land cover with lower mean AGB values, such as grasslands and pastures for example, were at higher risk of fire (Fig 5A and 5B). Accounting only for high fire risk area (>0.5 probability), potential mean AGB loss in the model 2010 output (0.88±0.3 Tg) was 85% higher than in the 2009 projection output (0.47±0.2 Tg) (Fig 5C and 5D).

**Fig 6:**
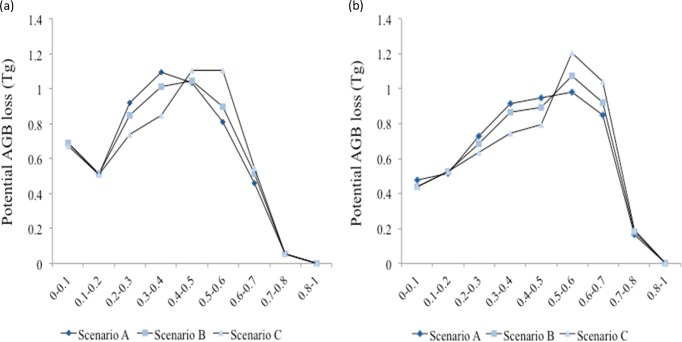
Mean potential aboveground biomass loss for each probability threshold of fire risk under the three scenarios (a) without climate change and (b) with climate change (using 2010 temperature and MCWD anomalies). Aboveground biomass was estimated averaging Yu et al. building on Saatchi et al. [92], Baccini et al. [93] and Mitchard et al. [94] datasets for the region and using Eq (1). Mean values need to be multiplied by ±0.096 to account for uncertainty in the range of AGB loss considered in Eq (1).

As expected, the potential AGB loss was higher in scenarios B and C than in scenario A. The rapid growth scenario C showed particularly high biomass loss in the probability range 0.5–0.7 (Fig 6). Considering only high fire risk area (> 0.5 probability), potential AGB loss in the scenario C (1.71±0.6 Tg) was 28% higher than in scenario A (1.33±0.5 Tg). Under drier climatic conditions, the potential AGB loss in high fire risk area increased even further with a loss of 2.00±0.6 Tg in scenario A and of 2.44±0.8 Tg in scenario C. The extreme scenario C under climate change had a potential AGB loss 1.8 times higher than the model 2010 output.

### Potential fire impacts on livelihoods

**Fig 7:**
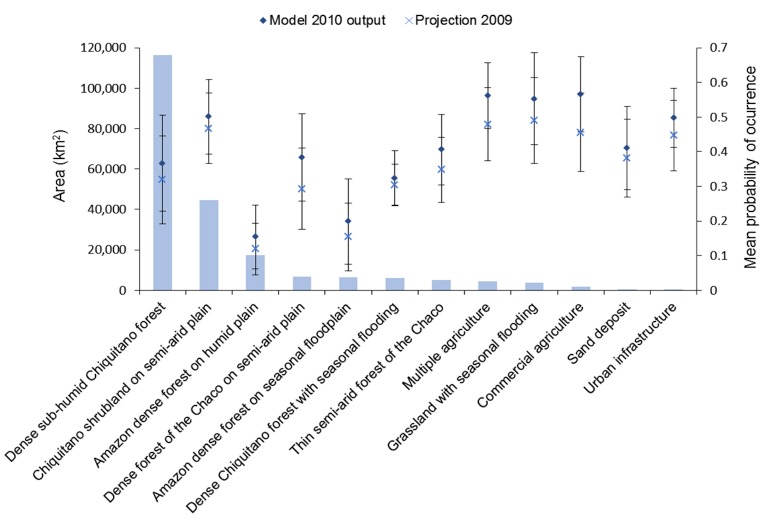
Bars show the total area covered by each category of the 2010 land use and land cover (LUC) map for the Chiquitania region, ranked by decreasing area. Points show the mean probability (±STD) of fire risk in different LUC categories for the model 2010 output and 2009 projection.

The largest land use and land cover (LUC) categories in the region were ‘dense sub-humid Chiquitano forest’ (116,300 km^2^) and ‘Chiquitano shrubland on semi-arid plain’ (44,700 km^2^). These categories were also potentially the most affected by fires in terms of area under risk. Focusing only on high fire risk (>0.5 probability), the area in the first category was 2 times larger in the model 2010 output (20,400 km^2^) than in the 2009 projection, and 45% larger in the second category with 25,600 km^2^ in the dry year. Dry conditions increased the mean probability of fire occurrence particularly in cleared areas with different types of land used for mixed agriculture (i.e. agriculture and cattle ranching), as well as in grasslands and the forest of the Chaco on semi-arid plain (Fig 7).

Across all scenarios, the two categories of the Land Use Plan (PLUS) with potentially most affected area by fires were ‘extensive cattle ranching’ and ‘forest use and regulated cattle ranching’. The most noticeable differences between scenario A and scenario C were in the ‘permanent forest production’ and ‘departmental protected area’ categories of the PLUS. In the scenario C, the area of high fire risk (>0.5 probability) doubled in the former category with 1,007 km^2^ and increased by 59% in the latter with 2,003 km^2^. Considering interactions with climate change, area at high fire risk more than doubled in the scenario C in the categories ‘forest use and regulated cattle ranching’ (37,400 km^2^), ‘permanent forest production’ (1,300 km^2^), and ‘intensive cattle ranching and agriculture’ (3,900 km^2^), increased by 89% in the category ‘departmental protected area’ (2,400 km^2^) and by 66% in the category ‘national protected area’ (24,400 km^2^) compared to scenario A without climate change. In general, comparisons between scenarios B and C showed less difference in high fire risk area in the ‘departmental protected area’ category. Hence, establishing new DPAs in the scenario A helped reduce fire risk in this PLUS category, even under severely dry conditions.

**Fig 8:**
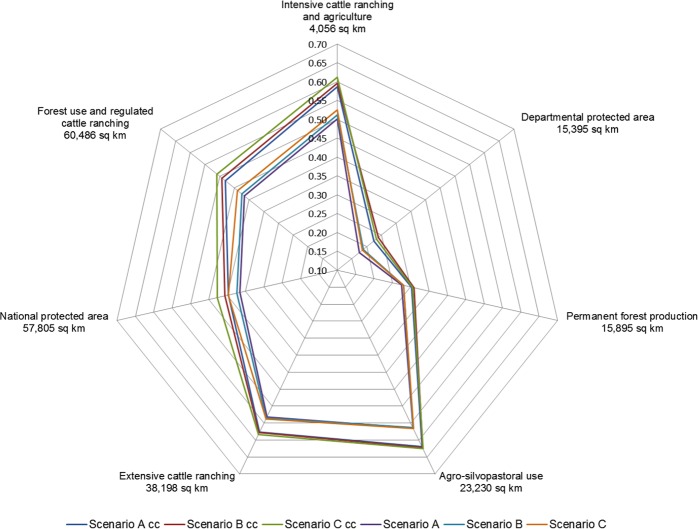
Mean probability of fire occurrence estimated for different categories of the Land Use Plan (PLUS) in the region for each simulation scenario A, B, and C, and considering climate change (cc). The area covered by the PLUS categories increases clockwise starting from the top of the radar.

The largest PLUS categories in the region were ‘forest use and regulated cattle ranching’ (60,500 km^2^) and the ‘national protected area’ (57,800 km^2^). These two categories showed the highest increase in mean probability of fire occurrence under the rapid growth scenario C (Fig 8). When considering also climate change the mean probability of fire occurrence increased across all scenarios. Mean probability values of fire occurrence reached about 0.6 in the categories ‘extensive cattle ranching’, ‘agro-silvopastoral use’ and ‘intensive cattle ranching and agriculture’ (Fig 8). These three categories are associated to cleared land with a mixture of pastures, fallow, and agriculture. Despite synergies between severely dry conditions and rapid frontier expansion increased the fire risk in categories ‘departmental protected area’ and ‘permanent forest production’, the mean probability of fire occurrence in these two categories remained relatively low compared to the other PLUS categories.

## Discussion

### Determinants of fire risk in the Chiquitania

We found that important determinants of fire risk are distance to roads, recent deforestation and density of human settlements. Other studies have also identified that forest fires are associated with road development and forest fragmentation, with the probability of fire decreasing as distance to roads and clearings increases [27,33,34,35,96]. This can partly be explained by fire escape from pastures or croplands that are being burned, but also as a result of drier conditions in forest edges [9]. Silvestrini *et al*. [35] found that forests located within 8 km of roads are highly vulnerable to fire in Amazonia, while Rodriguez-Montellano [51] estimated that 66% of forest fires in Bolivia occur within 1 km distance from deforested land.

In our model the dense network of secondary or unpaved roads was a better explanatory variable for fire risk than the paved roads. Although this is contrary to what was found by Gutiérrez-Vélez *et al*. [37], it corresponds to the local reality of the Chiquitania region where paved roads covered only 9% of the entire road network at the time of the study. Most of the local communities and properties were located along or close to unpaved secondary roads. We expect that the construction of more planned paved roads in the future will inevitably lead to more secondary roads, although we did not incorporate this effect in the simulations.

In our study recent deforestation (2000–2010) was more significant for fire risk prediction than consolidated deforestation (accumulated to 2000). This denoted strong connections between fire and burning for the conversion of forests to agricultural land. This was similar to results by Lima *et al*. [31] that showed a significant spatial association between recent deforestation and the occurrence of fires. Although in their study correlation between old deforestation and burn scars was low, they found a high number of burn scars in areas of old deforestation. In our study, old deforestation was removed from the model due to its low contribution to model gain, although it is important to note that we also found several hotspots in old deforestation areas. Aragão and Shimabukuro [11] showed that fire activity in Amazonia can be high where the rate of deforestation is lower because of burning to renew existing pasture areas (i.e. removing weeds and pests and promoting regeneration) or to clear vegetation regrowth for new pastures. Overall, the importance of recent deforestation to predict fire risk in our model is concerning, particularly when pondering future trajectories of rapid agricultural expansion encouraged by national policies.

Outmigration and emptying of rural landscapes was identified as an additional factor affecting fire frequency at a province-scale study conducted by Uriarte *et al*. [96] in western Amazonia. In the Chiquitania region, the density of human settlements was found to be an important factor contributing to fire risk. Contrary to the case study by Uriarte *et al*. [96], in the Chiquitania the new wave of immigration is increasing the rural population and expanding agricultural practices and fire use. Because fire is a cheap, labour-saving way of clearing and managing land, it is the technique adopted by most of the new farmers and cattle ranchers settling in the region, even if they do not use fire traditionally in their locations of origin. No prior traditional knowledge of fire use also represents an additional risk factor for accidental wildfires.

### Future fire risk and potential impacts

Our simulations showed that severely dry conditions increased the risk of fire in the Chiquitania region across all types of land use and land cover. Results also showed that the interactions between dry climatic conditions and rapid frontier expansion can further increase fire risk with potential negative implications in terms of carbon loss and livelihoods. A rapid growth scenario C with climate change presented an important potential biomass loss of 2.44±0.8 Tg in areas of high fire risk (>0.5 probability), which was 1.8 times higher than the estimates for the 2010 drought.

In addition, cleared areas used for agriculture and cattle ranching showed the highest mean probability of fire occurrence, which increased even further under drought conditions. These results have serious implications because they indicate that the three main subsistence and economic activities in the Chiquitania, i.e. the forestry, livestock, and agriculture sectors, are the most vulnerable to fire. Recent studies in western Amazonia [96,37] also found that drought severity significantly increases the risk of fire in cleared lands predominantly covered by agriculture and pastures.

Most of the agricultural production in the Chiquitania region is based on extensive production systems, which (i) depend on large pastures that according to Uhl and Kauffman [97] are the most flammable land cover susceptible to fire throughout most of the dry season, and (ii) are intertwined with secondary forests and fallow, which become more flammable with drought severity [37]. High mean probabilities (reaching up to 0.6 or more) of fire occurrence in PLUS categories ‘extensive cattle ranching’, ‘agro-silvopastoral use’ and ‘intensive cattle ranching and agriculture’ under rapid growth and extremely dry conditions means that fire management and wildfire risk reduction has to be at the core of land use and development policies promoting frontier expansion in the Chiquitania region.

### Inhibitory effects on fires

The factorial and exploratory analyses with wet and dry year datasets showed that the climatic variables became more important to predict probability of fire occurrence during dry years, and that dry conditions increased the susceptibility of forests to fire undermining their ability to inhibit or reduce risk. Although high fire risk areas remained close to the deforestation areas, along roads and in the agricultural zones, fire risk under extremely dry conditions became more widespread. Under severely dry conditions, high fire risk area (>0.5 probability) doubled in the ‘dense sub-humid Chiquitano forest’ and increased by 45% in the ‘Chiquitano shrubland’, which meant that the two largest categories of land cover in the region became more susceptible to fire. Even more, fire risk in our model spread into the northwestern area under 2010 drought conditions, affecting the more humid Amazon forests, which generally show a lower mean probability of fire occurrence and have higher capacity to inhibit fire. Similar results were observed by Silvestrini *et al*. [35] with 2050 simulations showing climate change alone may spread fire into the highly moist Amazon forests.

Maintaining large blocks of forests is recognised as critical for managing landscape-level fire in Amazonia, as extensive areas of forest can only be burned by many widely distributed fires given fire spread rates in the region [10]. This was demonstrated by studies in the Brazilian Amazonia, which found that the network of protected areas was effective at limiting the spread of forest fires [73,35,74]. However, a very recent study by Carmenta *et al*. [83], which evaluated fire activity in and around 49 Sustainable Use Reserves in Brazil, found that reserve creation itself had no impact on spatial fire density or improved fire management (i.e. burning time). Their study demonstrated that the effectiveness of reserve areas to protect forests from wildfires is not necessarily due to management but actually due to their location in more remote and sparsely inhabited areas (i.e. *de facto* differences between the protected areas and unprotected areas). This highlights the importance of assessing the impact of PA creation in the context of pre-existing landscape attributes.

In the CMF we observed that only the Departmental Protected Areas (DPAs, and TIOCs located in DPAs) had the ability to inhibit fire risk. Under the sustainability scenario A, the establishment of DPAs in areas of high fire risk helped reduce probability of fire occurrence, even under severely dry conditions. We think this can be explained by a combination of *de facto* and institutional factors. Location and pre-existing landscape attributes are most likely the main factors explaining these results because existing DPAs (used to train the model) were mainly located in northern Chiquitania where forests were more humid and less prone to fire, and road and population densities were lower.

Nevertheless, differences in priorities and institutional settings that determine management of protected areas may also have influenced the effectiveness of DPAs to inhibit fire risk. A comparison with national PAs provides insights to elaborate on this point. Similarly to DPAs, we found that national protected areas were for the most part located in areas with lower road and population densities. Yet they showed more effect on the probability of fire occurrence than DPAs. The higher fire risk in national protected areas may partly relate to their larger size but we also think it may be associated to their designation type, which involved mainly a combination of national parks and natural areas of integrated management. The latter allows human settlements and production activities within the area, a factor that can contribute to escaped/accidental fires within the PAs. Furthermore, national protected areas depend on the central government, whilst DPAs in the region are managed by the Autonomous Departmental Government of Santa Cruz. The regional government has employed an increasing number of park rangers to monitor its network of protected areas after 2010, and has invested in capacity for fire management and control practices (*pers obs)*.

When merging all the protected areas and indigenous land into fewer categories (i.e. protected areas, indigenous land, and combined) we observed that the only category that showed an inhibitory effect was the combined PA and TIOC category. This means that TIOCs also seemed to play a role in protecting forests from wildfires, and indicates a need for further research. Improving the performance of PAs/TIOCs to be effective inhibitors of fire risk would require more contextual information that can help better differentiate the contributing factors, including (i) type of designation/protection and management that could be most effective, (ii) fire use practices and human activities that should be allowed within the area, (iii) the types of fire observed in the areas, (iv) the pre-protection baseline to compare more objectively, and (v) the landscape configuration within and surrounding the protected areas.

In relation to landscape configuration, Gutiérrez-Vélez *et al*. [37] found that in western Amazonia local landscape homogeneity increased fire spread while discontinuities in heterogeneous landscapes acted as firebreaks. On the contrary, other studies [98,36] identified forest fragmentation and landscape heterogeneity as main drivers increasing susceptibility of tropical forests to fire, thus suggesting landscape homogeneity as critical for managing landscape-level fire risk. Also, suppressing the use of fire within extended tropical forest areas intertwined with grasslands, such as is the case in the Chiquitania, has been observed to lead to increased vulnerability to even larger wildfires in other studies [99–102]. This clearly emphasizes the need to further study the right balance between landscape patchiness and homogeneity and the combination of land management and fire use safeguards that should be allowed within and around DPAs, TIOCs and other PA categories for them to be more effective wildfire inhibitors.

All in all, we must recognise that protected areas and indigenous land did not contribute significantly to model gain in this study. Despite DPAs showed promising inhibitory effects worth analysing further, it is important to bear in mind that the establishment of protected areas can be challenged by the socio-institutional context that will ultimately influence their function, or even feasibility. In addition, while protected areas may be helpful in addressing wildfire spread and reducing ignitions within a particular area, this would not necessarily deal with (i) the causes and main determinants of fire occurrence identified in this paper, (ii) the spreading use of fire as population and agricultural production continue to grow, and (iii) the leakage that protecting an area may cause in other unprotected areas of the landscape. With these concerns in mind, the establishment of PAs as a wildfire risk management strategy should be thought as complementary to other wildfire risk strategies, and as a case for testing and learning more about contextual factors (i.e. landscape and social attributes) that can improve landscape-level fire risk management.

### The fire risk model as a decision-support tool

This study demonstrated that a probabilistic modelling approach using MaxEnt is appropriate to study the fire-climate-society nexus generating insights about future wildfire risk based on anthropogenic, biophysical and climatic determinants. Moreover, we believe that this simpler fire risk modelling approach increases the potential for the model to be used as a decision-support tool. The model can be easily updated on an annual basis using inputs from already existing systems that monitor annual land cover change and track hotspots. These systems are coordinated by the government, as well as by local research institutes, but are currently poorly integrated. In this sense, the model could even serve as a ‘boundary object’ [103] to integrate different types of data and information generated by these systems in a way that improves collaboration between the different agencies, and increases their capacity to anticipate and manage increased wildfire risk in the future. Star and Griesemer [104] defined ‘boundary object’ as an analytic concept that has different meanings in different social worlds but with a structure that is common enough to more than one world to make it recognizable. Cash *et al*. [103] recognised the translation purpose of this concept and the potential of boundary objects–such as models–to help disparate perspectives come together and eventually co-produce information that can be more salient, credible and legitimate for decision-making.

Further research to improve model performance and understanding of wildfire dynamics in the Chiquitania region could focus on including land tenure to differentiate the effects of large-scale and small-scale landholders. A model with even finer resolution could capture in more detail understory fire from slash-and-burn activities and pasture burning in smaller fields, but it would require a different source of samples to calibrate the model and a smaller area to focus on. Early developments currently increasing the resolution of remotely-sensed data for fire monitoring could be used to this end, such as the combined Landsat-8 and Sentinel-2 burnt area product with a 10 to 60 m multi-spectral global coverage [105] and the new Visible Infrared Imaging Radiometer Suite (VIIRS) active fire detection algorithm generating improved 375 m imagery data [106]. Understanding micro-scale behaviour could be particularly relevant to study the effects of bottom-up strategies to manage wildfire, which could be integrated with agent-based modelling.

## Conclusions

Anticipating increased fire risk in the future is crucial given plans to expand the agricultural frontier and predictions of more extreme dry seasons in the Chiquitania. Severely dry conditions like the 2010 drought showed to increase fire risk across all land use and land cover categories with 85% more potential biomass loss in areas of high fire risk compared to a normal/wet year like 2009. This risk was even higher when drought conditions interacted with rapid land cover change. Land used for agriculture and cattle ranching seemed particularly vulnerable to fire occurrence under these conditions, highlighting the need for wildfire risk management to be at the core of land use and development policies, and the importance of anticipatory planning to prevent potential impacts associated to large wildfires in the future. Departmental protected areas (and TIOCs located within) showed potential inhibitory effects, but further research and monitoring efforts would need to identify the contextual factors and appropriate land management strategies that could improve their effectiveness to reduce wildfire risk.

This novel and simple modelling approach based on maximum entropy to simulate different scenarios of fire risk has shown potential as a support tool to inform land and fire management decisions at the regional level. The model can be easily updated with inputs from already existing, albeit fragmented, systems that monitor anthropogenic activities and active fires in Bolivia. Using the model to gain foresight of future risk can help identify management strategies that deal with uncertainty and account for interactions between development trajectories and climatic conditions. The approach is easy to replicate in other tropical landscapes that are facing a transition to disturbance regimes dominated by more frequent and larger wildfires.

## Supporting Information

S1 Fig Dry season severity profile averaged for the Chiquitania region using the 3-month Standardized Precipitation Index (SPI-3) from Jan-Feb-March 2000 to Oct-Nov-Dec 2013. The year 2010 shows a particularly prolonged low SPI-3. The SPI is the number of standard deviations that the observed cumulative precipitation during any given period of interest deviates from the climatological average. SPI data were obtained from the NASA GPCP V2 in the IRI Data Library. Available: http://iridl.ldeo.columbia.edu/SOURCES/.IRI/.Analyses/.SPI/.SPI-CAMSOPI_3-Month/.
(JPG)

S2 Fig Selected non-climatic variables for the wildfire risk model, involving (a) deforestation from 2000 to 2010, (b) land use and land cover updated to 2010, (c) human settlements, unpaved (secondary) roads and paved (primary) roads updated to 2010, and (d) different categories of protected area (PA) and indigenous land (TIOC) consolidated by 2009.
(JPG)

S3 Fig Selected climate-related variables for the wildfire risk model, involving temperature anomalies for (a) 2009 and (b) 2010 estimated using the baseline mean temperature for the period 2000–2010, and maximum climatological water deficit (MCWD) anomalies for (c) 2009 and (d) 2010 estimated using the baseline mean MCWD for the period 2000–2010.
(JPG)

S4 Fig Maps showing changes in (a) paved and unpaved roads, (b) different protected areas and indigenous land categories, and (c) deforestation assumed for sustainability scenario A, business as usual scenario B, and rapid growth scenario C.
(JPG)

S5 Fig Bars show the probability of fire occurrence associated to each category of the variable that combines protected area (PA) and indigenous land (TIOC). Results are obtained from running the ‘model 2010’ with this variable only. The categories of the variable are: (1) no PA, no TIOC, (2) National PA, (3) Departmental PA, (4) Municipal PA, (5) TIOC only, (6) TIOC in National PA, (7) TIOC in Departmental PA, and (8) TIOC in Municipal PA.
(JPG)

S6 Fig Results of the jackknife test of variable importance for the ‘model 2010’. The variable with highest gain when used in isolation is ‘*roads*’ (roadcom, road network weighted by paved and unpaved roads), which appears to have the most useful information by itself. The variable that decreases the gain the most when it is omitted is ‘*Chiquitano shrubland*’ (cobuso_31), which appears to have the most information that is not present in the other variables. Values shown are averages over replicate runs. Other variables are: ‘*deforestation*’ (defp, deforestation between 2000 and 2010), ‘*population density’* (comden_mpo, density of human settlements weighted by population in each Municipality), ‘*temperature*’ (tanoms, mean temperature anomalies), ‘grasslands’ (cobuso_34), ‘*precipitation*’ (panoms, maximum climatological water deficit (MCWD) anomalies), and ‘*protected areas*’ (patco, which includes different categories of protected areas and indigenous land).
(JPG)

S7 Fig 2009 MCD14ML hotspot density across different probability thresholds of the 2009 projection generated with the ‘model 2010’. (JPG)

S1 File Advantages and limitations of maximum entropy (MaxEnt) modelling for fire risk. (PDF)

S2 File Data source and processing. (PDF)

S3 File Additional information on Eq (1) used to estimate potential aboveground biomass (AGB) loss. (PDF)

S1 Table Variables tested individually and in different combinations for fire risk modelling. (PDF)

S2 Table Categories of the Land Use Plan (PLUS) of the Department of Santa Cruz processed for the Chiquitano Model Forest region. (PDF)
