# Supplementary figures and images for: Increased Wildfire Risk Driven by Climate and Development Interactions in the Bolivian Chiquitania, Southern Amazonia
Source: PLoS One. 2016 Sep 15;11(9):e0161323. doi: 10.1371/journal.pone.0161323 (PMC5025183; doi:10.1371/journal.pone.0161323)

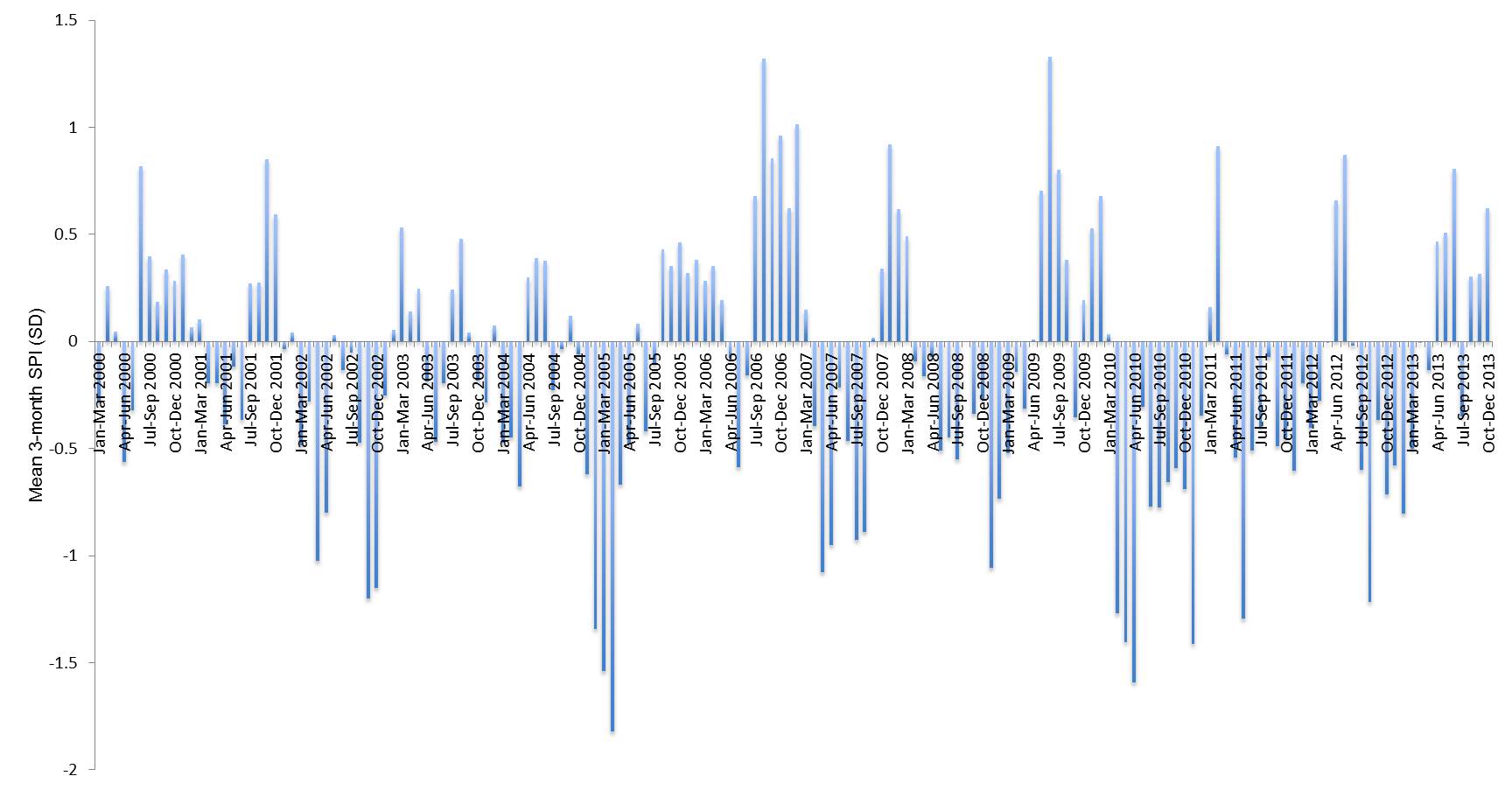

Supplement: S1 Fig — The year 2010 shows a particularly prolonged low SPI-3. The SPI is the number of standard deviations that the observed cumulative precipitation during any given period of interest deviates from the climatological average. SPI data were obtained from the NASA GPCP V2 in the IRI Data Library. Available: http://iridl.ldeo.columbia.edu/SOURCES/.IRI/.Analyses/.SPI/.SPI-CAMSOPI_3-Month/. (JPG) [file pone.0161323.s001.jpg]

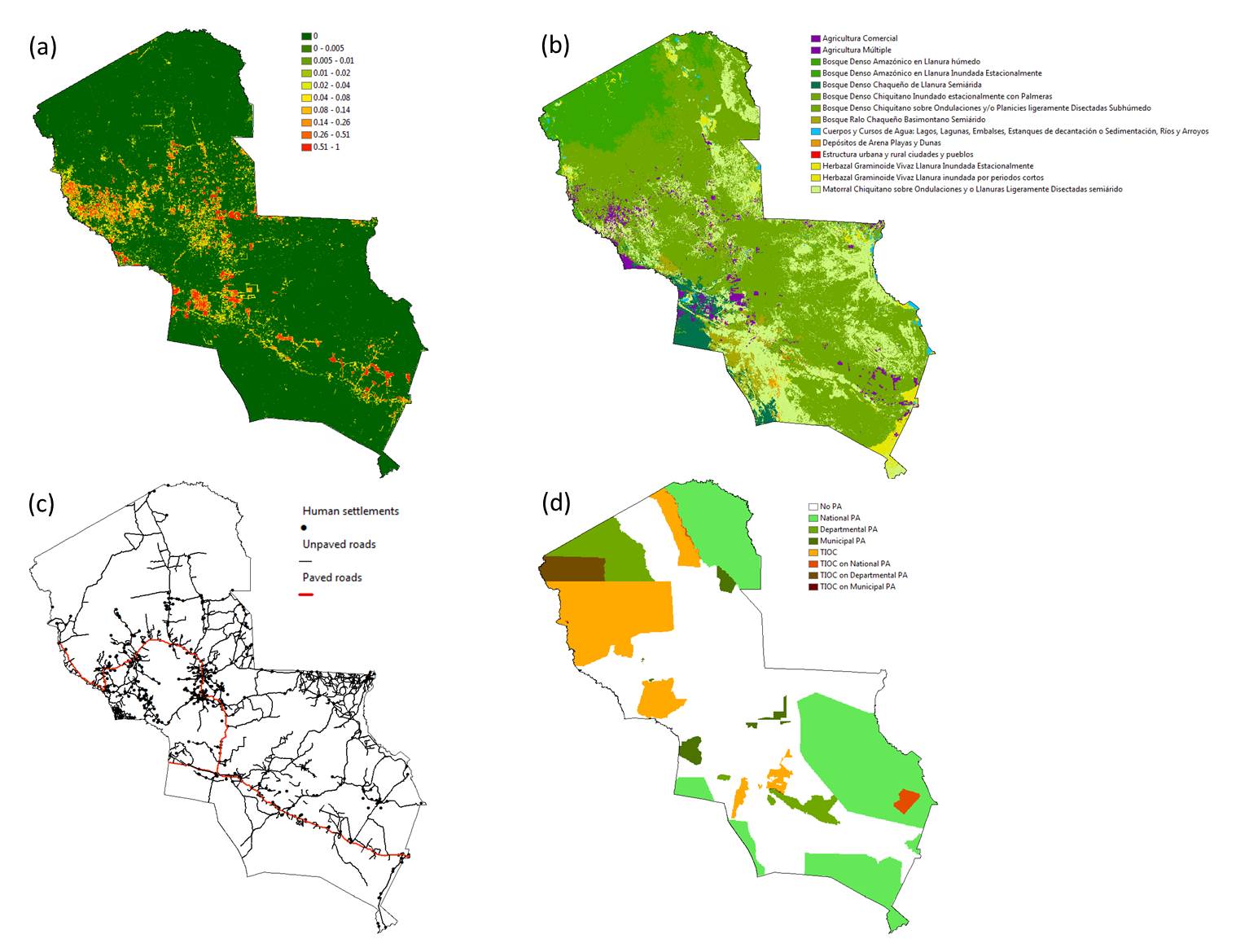

Supplement: S2 Fig — Selected non-climatic variables for the wildfire risk model, involving (a) deforestation from 2000 to 2010, (b) land use and land cover updated to 2010, (c) human settlements, unpaved (secondary) roads and paved (primary) roads updated to 2010, and (d) different categories of protected area (PA) and indigenous land (TIOC) consolidated by 2009. (JPG) [file pone.0161323.s002.jpg]

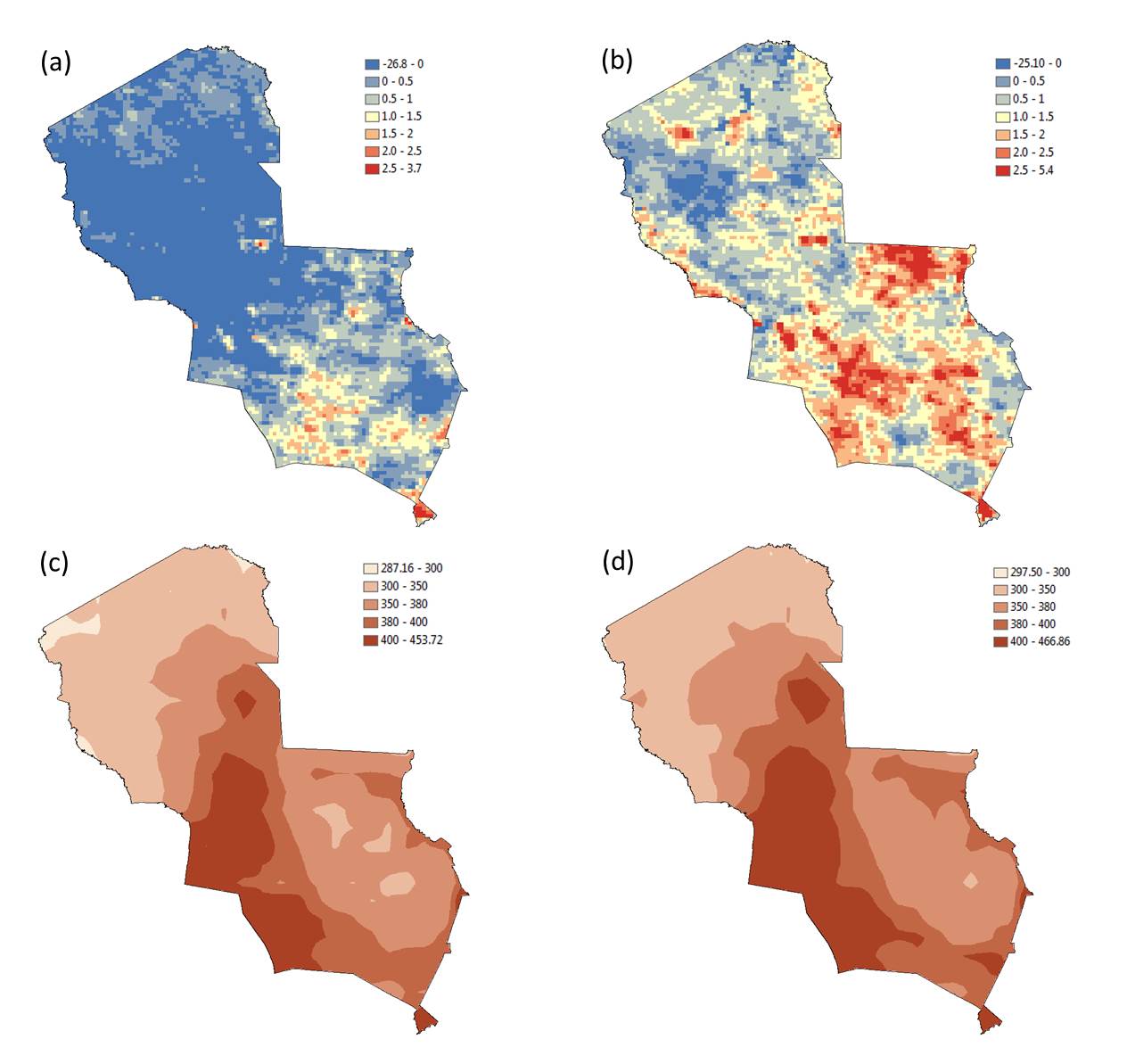

Supplement: S3 Fig — Selected climate-related variables for the wildfire risk model, involving temperature anomalies for (a) 2009 and (b) 2010 estimated using the baseline mean temperature for the period 2000–2010, and maximum climatological water deficit (MCWD) anomalies for (c) 2009 and (d) 2010 estimated using the baseline mean MCWD for the period 2000–2010. (JPG) [file pone.0161323.s003.jpg]

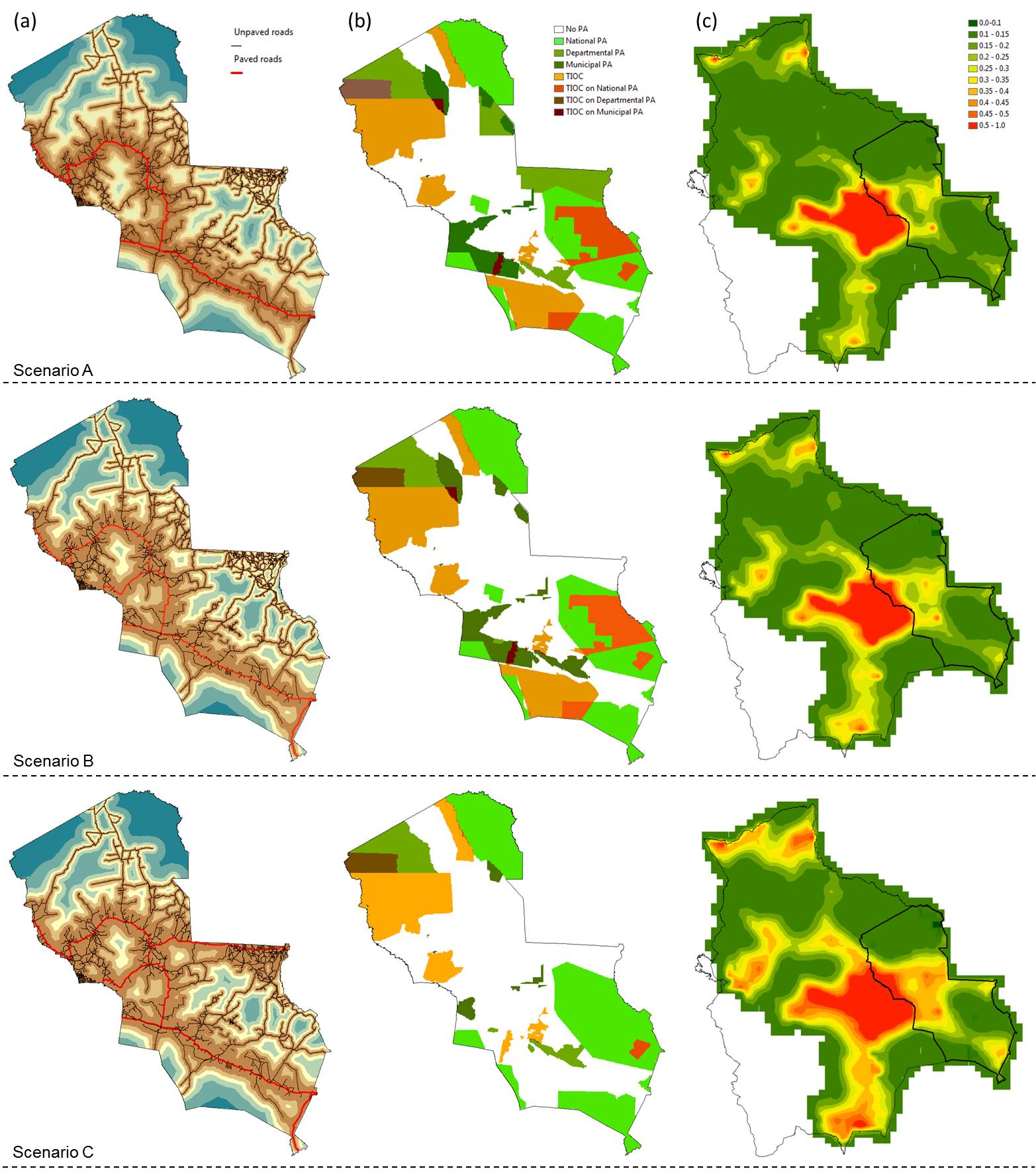

Supplement: S4 Fig — Maps showing changes in (a) paved and unpaved roads, (b) different protected areas and indigenous land categories, and (c) deforestation assumed for sustainability scenario A, business as usual scenario B, and rapid growth scenario C. (JPG) [file pone.0161323.s004.jpg]

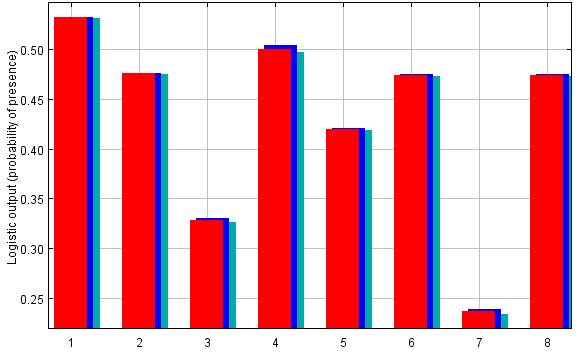

Supplement: S5 Fig — Results are obtained from running the ‘model 2010’ with this variable only. The categories of the variable are: (1) no PA, no TIOC, (2) National PA, (3) Departmental PA, (4) Municipal PA, (5) TIOC only, (6) TIOC in National PA, (7) TIOC in Departmental PA, and (8) TIOC in Municipal PA. (JPG) [file pone.0161323.s005.jpg]

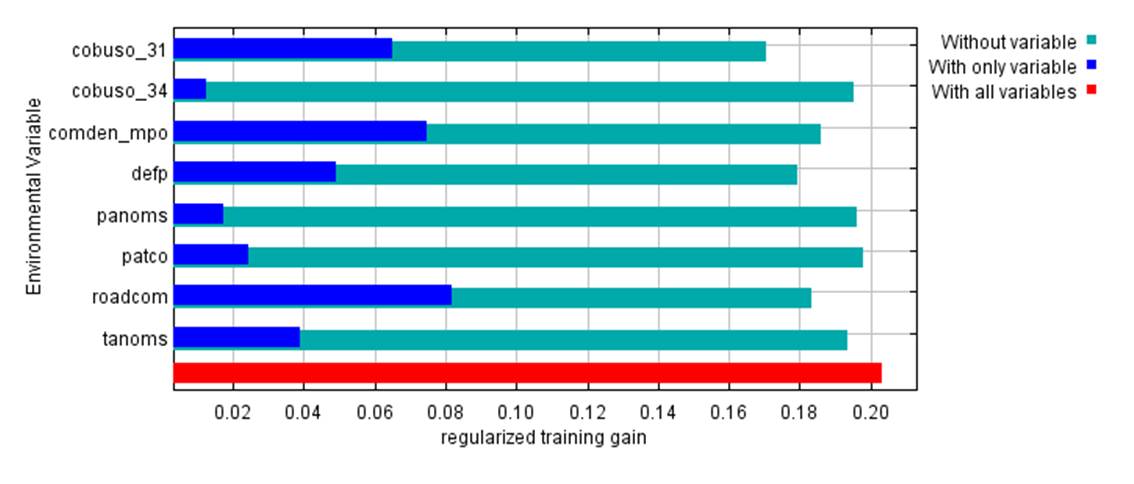

Supplement: S6 Fig — The variable with highest gain when used in isolation is ‘roads’ (roadcom, road network weighted by paved and unpaved roads), which appears to have the most useful information by itself. The variable that decreases the gain the most when it is omitted is ‘Chiquitano shrubland’ (cobuso_31), which appears to have the most information that is not present in the other variables. Values shown are averages over replicate runs. Other variables are: ‘deforestation’ (defp, deforestation between 2000 and 2010), ‘population density’ (comden_mpo, density of human settlements weighted by population in each Municipality), ‘temperature’ (tanoms, mean temperature anomalies), ‘grasslands’ (cobuso_34), ‘precipitation’ (panoms, maximum climatological water deficit (MCWD) anomalies), and ‘protected areas’ (patco, which includes different categories of protected areas and indigenous land). (JPG) [file pone.0161323.s006.jpg]

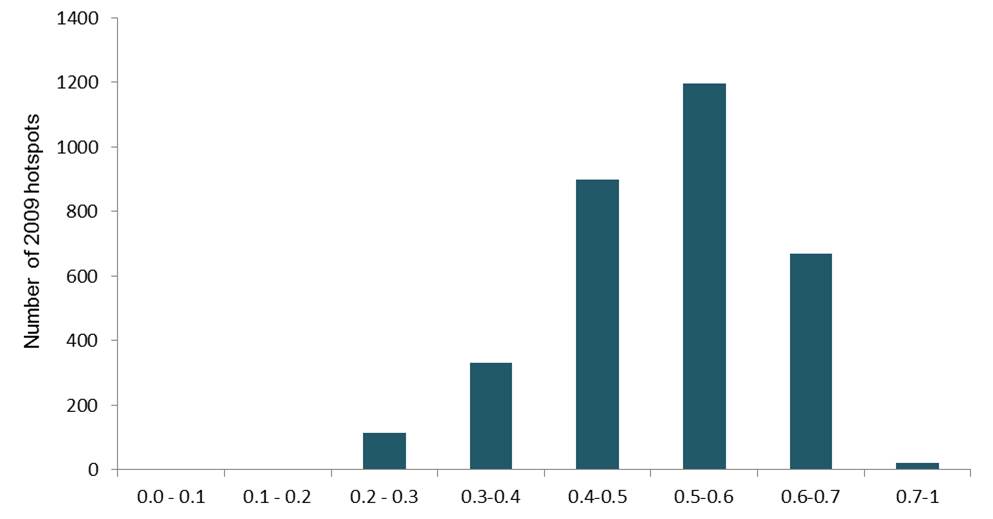

Supplement: S7 Fig — (JPG) [file pone.0161323.s007.jpg]
