# Supplementary material for: Increased Wildfire Risk Driven by Climate and Development Interactions in the Bolivian Chiquitania, Southern Amazonia
Source: PLoS One. 2016 Sep 15;11(9):e0161323. doi: 10.1371/journal.pone.0161323 (PMC5025183; doi:10.1371/journal.pone.0161323)
Supplement: S1 File — (PDF) [file pone.0161323.s008.pdf]

Advantages

First, MaxEnt uses only presence data, recognising that absence data are rarely available or reliable (Phillips et al. 2006; Elith et al. 2011; Phillips and Elith 2013). This is appropriate for species distribution modelling concerned with predicting areas of potential species occurrence – for which MaxEnt has been largely used – but also for modelling potential wildfire risk since fires do not occur in all places where fire-prone conditions exist, and it is difficult to define a true absence when monitoring fire (particularly when using hotpixels as samples to calibrate the model). With MaxEnt, background values in the environment where fire has not been observed are not treated as absences during the modelling task (Renard et al. 2012; Arnold et al. 2014). Second, the model output (i.e. maximum likelihood estimate of relative probability of presence) is continuous allowing for fine distinctions to be made between the levels of wildfire risk in different areas. This helps to make a more nuanced interpretation than presence-absence predictions (Phillips et al. 2006; Arnold et al. 2014). Third, MaxEnt is a generative approach that uses the environmental data from across the study area rather than a discriminative approach, which is an advantage when presence data is limited (Phillips and Elith 2013).

Limitations

There are some drawbacks with using maximum entropy modelling that most recent developments are trying to address. One main limitation is the possibility of over-fitting, limiting the capacity of the model to generalize well to independent data. The 'regularization multiplier' parameter in MaxEnt aims to address this by limiting the complexity of the model and generating a less localized prediction (Phillips and Dudík 2008). Another important pitfall that affects the accuracy of presence-only modelling relates to biases in the occurrence localities. In fire risk models, biases can occur depending on the fire dataset used. Using remotely sensed datasets rather than field-based observations can somewhat address location bias by providing a more complete dataset available for the study area and reducing sampling bias (Arnold et al. 2014). However, we acknowledge that biases with remotely sensed datasets also exist. Biases with MODIS-detected (MCD14ML) hotspots used as samples to calibrate the model can happen due to fire that started and ended between satellite overpasses, fires that are too small or cool to be detected by the MODIS footprint, cloud cover, heavy smoke, or tree canopy that may completely obscure fires such as in the case of small understory fires (Giglio 2010).

References

Arnold JD, Brewer SC, Dennison PE. Modeling climate-fire connections within the Great basin and Upper Colorado River Basin. Fire Ecology. 2014;10(2): 64–75.

Elith J, Phillips SJ, Hastie T, Dudík M, Chee YE,Yates CJ. A statistical explanation of MaxEnt for ecologists. Diversity and Distributions. 2011;17: 43–57.

Giglio L. MODIS Collection 5 Active Fire Product User's Guide. Version 2.4. 2010. University of Maryland, Maryland, US.

Phillips SJ, Anderson RP, Schapire RE. Maximum entropy modelling of species geographic distributions. Ecological Modelling. 2006;190: 231-259.

Phillips SJ, Dudík M. Modeling of species distributions with MaxEnt: new extensions and a comprehensive evaluation. Ecography. 2008;31: 161–175.

Phillips JS, Elith J. On estimating probability of presence from use-availability or presence-background data. Ecology. 2013;94(6): 1409-1419.

Renard Q, Pélissier R, Ramesh BR, Kodandapani N. Environmental susceptibility model for predicting forest fire occurrence in the Western Ghats of India. International Journal of Wildland Fire. 2012;21: 368–379.
