# Supplementary material for: Increased Wildfire Risk Driven by Climate and Development Interactions in the Bolivian Chiquitania, Southern Amazonia
Source: PLoS One. 2016 Sep 15;11(9):e0161323. doi: 10.1371/journal.pone.0161323 (PMC5025183; doi:10.1371/journal.pone.0161323)
Supplement: S2 File — (PDF) [file pone.0161323.s009.pdf]

The ‘*land cover*’ variables were processed using the official land use and land cover map for Bolivia updated to 2010 at 50m resolution developed by the National Technical Unit of Land Information (UTNIT) using Landsat 5 TM imagery. This map contains 14 categories for the region. Only 5 of these categories showed significant contribution to model gain: commercial agriculture, multiple agriculture, grassland, Chiquitano shrubland, and Chiquitano dry forest. After running multiple models to test different combination of variables and reduce variables with redundant information and low contribution to model gain, we selected only 2 land cover categories for the final models, namely Chiquitano shrubland and grassland.

The ‘*deforestation*’ variable was processed using the deforestation map developed by the Fundación Amigos de la Naturaleza (FAN) at 30 m resolution. This included deforestation accumulated to 2000, between 2000 and 2005 and between 2005 and 2010. We processed the data to obtain percentage of deforestation in 1 km pixel. We tested in the model the deforestation accumulated to 2000 (consolidated deforestation) and deforestation between 2000 and 2010 (more recent deforestation). The latter was selected for the final models due to its high contribution to model gain.

The ‘*roads*’ variable was processed using the road network map developed by the Fundación para la Conservación del Bosque Chiquitano (FCBC) updated to 2008 based on data from the Bolivian Road Network Administrator (ABC). The map showed 7 categories of roads including roads of the Fundamental Network, Prefectural network, Municipal network, and other secondary road types. Paved and unpaved roads were separated according to the ABC public information on the Fundamental Network projects concluded by 2014 and planned by 2020. The road variables processed and tested in the modelling task were Euclidian distance (i.e. distance between each map cell to the closest map cell of the target feature) to paved roads, to unpaved roads, and to all roads weighted by paved and unpaved roads. The latter was used for the final models as it combined the different types of roads and showed a high contribution to model gain.

The ‘*population density’* variable was processed using point data on human settlements developed by the FCBC updated to 2008 and population data per Municipality projected to 2010 obtained from the National Institute of Statistics (INE 2010). Density of human settlements was estimated using kernel density weighted by the population of each Municipality. We accounted for the fact that some four Municipalities (i.e. Charagua, Asención de Guarayos, El Puente and Pailón) are only partially included in the CMF region, so we calculated the percentage of population in the Municipality present in the region using population information by community gathered in the 2001 national census by the INE, and used this information to update the variable of human density weighted by population used in the modelling.

The '*protected areas'* variable was processed using data generated by the FCBC and the National Service of Protected Areas (SERNAP) updated to 2011. Protected areas (PAs) have different categories including integral protection where exploitation of natural resources is not allowed (e.g. natural parks, wildlife sanctuaries, etc.) and categories where sustainable use and management of natural resources is permitted (e.g. integrated management natural area, wildlife reserve, etc). In addition, protected areas can be established and managed at different scales, from national to municipal, with associated implications on resources and capacity to manage and monitor them. Besides PAs, in Bolivia there are also areas recognised as indigenous territory referred to since 2011 as *Territorio Indigena Originario Campesino* (TIOC). The State first recognized these areas in the 1990s, and many are currently consolidated with land title. In these areas indigenous people have the right to land and natural resources management according to their traditions, culture and organisation (Chumacero et al. 2010). In the lowlands of Bolivia, the TIOCs occupy large extensions and include primary forests in locations of lower road connectivity. Thus, they have an important role to play in forest conservation if managed sustainably (Müller et al. 2013). For modelling we combined the PAs and the TIOCs consolidated by 2009 into a single variable with 8 categories: no protection, national PA, departmental PA, municipal PA, TIOC, TIOC in a national PA, TIOC in a departmental PA, and TIOC in a municipal PA.

The '*temperature*' variable was processed using monthly land surface temperature (LST) data from MODIS Terra (MOD11C3) at 0.05^{o} for the period 2000 to 2010 filtered only for day data. For each year mean annual temperature was calculated given that intra-annual temperature variation was small. Temperature anomalies for each year were estimated using the 2000-2010 mean temperature as baseline. Different techniques were used to correct the anomalies for land cover change in the decade, which we recognise can affect LST. The technique that generated best results for modelling was to normalise the 2010 and 2009 temperature anomalies using the 2004 temperature anomalies (i.e. a wet normal year similar to 2009 with anomalies that overlap with most of the deforestation 2000 to 2010).

The '*precipitation*' variable was processed using monthly rainfall data at 0.25^{o} for the period 2000 to 2010 from the Tropical Rainfall Measuring Mission (TRMM). Climatological water deficit (CWD) and maximum climatological water deficit (MCWD) were calculated for each year in this period applying a threshold of 100 mm to the dataset as conducted for other studies in this region and Amazonia (Malhi et al. 2009; Lewis et al. 2011; Doughty et al. 2015). The mean evapotranspiration rate of a moist tropical rainforest is about 100 mm per month (Malhi and Wright 2004). Therefore, it is estimated that the forest is in net water deficit when the precipitation (P) is less than 100 mm per month. This threshold is commonly used to define dry season, and a common parameter for dry-

season length is the number of months per year with *P* <100 mm. Malhi and Wright (2004) pointed out that CWD is only an approximate indicator of actual soil water deficit. We calculated MCWD anomalies for each year using the 2000-2010 mean MCWD as baseline. We applied bilinear resampling technique to smooth the coarse-resolution MCWD data and anomalies and obtain the 1 km resolution used for the modelling task.

The *hotspots* used as samples to calibrate the models corresponded to MODIS Aqua and Terra MCD14ML hotspots for the period 2001-2010 (product version 5.1) obtained from the Fire Information for Resource Management System (FIRMS 2014). The hotspots were filtered for high confidence >80% (Giglio 2010) to minimize false alert in predictions.

References

Doughty, CE, Metcalfe DB, Girardin CAJ, Amezquita FA, Cabrera DG, Huasco WH, Silva-Espejo JE, Araujo-Murakami A, da Costa MC, Rocha W, Feldpausch TR, Mendoza ALM, da Cost ACL, Meir P, Phillips OL, Malhi Y. Drought impact on forest carbon dynamics and fluxes in Amazonia. Nature. 2015; 519: 78–82.

Chumacero JP, Tinta E, Salgado J, Vadillo A, Colque G, Ortiz MV, Calizaya O, Costas P. Fundación Tierra. Informe 2010, Territorios Indígena Originario Campesinos en Bolivia-Entre la Loma Santa y la Pachamama. 2010. La Paz, Bolivia.

Giglio L. MODIS Collection 5 Active Fire Product User's Guide. Version 2.4. 2010. University of Maryland, Maryland, US.

INE. Instituto Nacional de Estadística de Bolivia. 2010 [accessed October 2012] Available: http://geo.ine.gob.bo/cartografia/

Lewis SL, Brando PM, Phillips OL, van der Heijden GMF, Nepstad D. The 2010 Amazon drought. Science. 2011;331: 554.

Malhi Y, Wright J. Spatial patterns and recent trends in the climate of tropical rainforest regions. Phil. Trans. R. Soc. Lond. B. 2004; 359: 311–329.

Malhi Y, Aragao LEOC, Galbraith D, Huntingford C, Fisher R, Zelazowski P, Sitch S, McSweeney C, Meir P. Exploring the likelihood and mechanism of a climate-change-induced dieback of the Amazon rainforest. Proceedings of the National Academy of Sciences of the United States of America. 2009;106: 20610–20615.

Müller R, Pistorius T, Rohde S, Gerold G, Pacheco P. Policy options to reduce deforestation based on a systematic analysis of drivers and agents in lowland Bolivia. Land Use Policy. 2013;30(1): 895-907.

NASA FIRMS. FIRMS. Fire Information for Resource Management System. 2014 [accessed November 2014]. Available: https://earthdata.nasa.gov/earth-observation-data/near-real-time/firms
