# Supplementary material for: Increased Wildfire Risk Driven by Climate and Development Interactions in the Bolivian Chiquitania, Southern Amazonia
Source: PLoS One. 2016 Sep 15;11(9):e0161323. doi: 10.1371/journal.pone.0161323 (PMC5025183; doi:10.1371/journal.pone.0161323)
Supplement: S3 File — (PDF) [file pone.0161323.s010.pdf]

The linear Eq. (1) for AGB loss assumes that the remaining AGB in an area affected by fire is strongly correlated with the initial AGB before fire, which is coherent with the expectation that as biomass increases the microclimate below the canopy tends to become wetter and cooler reducing the intensity and suitability for fire spread (Brando et al. 2012). The proportion range of AGB loss due to fire is based on results from 13 studies conducted in forest plots across Amazonia one year after the fire occurrence (Anderson et al. 2015), and forest plots located in the Chiquitania region where AGB loss was estimated five years after fire occurrence (Devisscher et al. 2016). The range is in line with other studies that have used a post-fire tree mortality rate of 10-50% depending on fire intensity and state of the forest (Alencar et al. 2006).

References

Alencar, AAC, Nepstad DC, Vera Diaz MdC. Forest understory fire in the Brazilian Amazon in ENSO and non ENSO years: area burned and committed carbon emissions. Earth Interactions. 2006;10: 1–16.

Anderson LO, Aragão LEOC, Gloor M, Arai E, Adami M, Saatchi S, Malhi Y, Shimabukuro YE, Barlow JB, Berenger E, Duarte V. Disentangling carbon emissions due to fires in southern Amazonia during the 2010 drought. Global Biogeochemical Cycles. 2015;29: doi:10.1002/2014GB005008.

Brando PM, Nepstad DC, Balch JK, Bolker B, Christman MC, Coe M, Putz FE. Fire-induced tree mortality in a neotropical forest: the roles of bark traits, tree size, wood density and fire behavior. Global Change Biology. 2012;18: 630–641.

Devisscher T, Malhi Y, Rojas Landívar VD, Oliveras I. Understanding ecological transitions under recurrent wildfire: A case study in the seasonally dry tropical forests of the Chiquitania, Bolivia. 2016;360: 273-286.
