# Supplementary material for: Increased Wildfire Risk Driven by Climate and Development Interactions in the Bolivian Chiquitania, Southern Amazonia
Source: PLoS One. 2016 Sep 15;11(9):e0161323. doi: 10.1371/journal.pone.0161323 (PMC5025183; doi:10.1371/journal.pone.0161323)
Supplement: S1 Table — (PDF) [file pone.0161323.s011.pdf]

Variable	Original resolution	Source
Global Human Influence Index	1 km	Wildlife Conservation Society - WCS, and Center for International Earth Science Information Network - CIESIN - Columbia University 2005. Last of the Wild Project, Version 2, 2005 (LWP-2): Global Human Influence Index (HII) Dataset (Geographic). Palisades, NY: NASA Socioeconomic Data and Applications Center (SEDAC). http://dx.doi.org/10.7927/H4BP00QC.
Global forest cover loss (2000-2012)	30 m	Hansen, M. C., P. V. Potapov, R. Moore, M. Hancher, S. A. Turubanova, A. Tyukavina, D. Thau, S. V. Stehman, S. J. Goetz, T. R. Loveland, A. Kommareddy, A. Egorov, L. Chini, C. O. Justice, and J. R. G. Townshend 2013. "High-Resolution Global Maps of 21st-Century Forest Cover Change." Science 342: 850–53.
Land use and land cover map for Bolivia (all categories and each category individually)	50 m	National Technical Unit of Land Information (UTNIT) 2011.
Deforestation accumulated to 2010, deforestation accumulated to 2000, and deforestation 2000-2010 (as separate variables)	30 m	Fundación Amigos de la Naturaleza (FAN) 2012.
Human settlements	n/a	Fundación para la Conservacion del Bosque Chiquitano (FCBC) 2008.
Population density (combining human settlements and population density by Municipality)	n/a	Fundación para la Conservacion del Bosque Chiquitano (FCBC) 2008, National Institute of Statistics (INE 2010).
Roads (all roads, separating paved and unpaved, combining and weighting roads)	n/a	Fundación para la Conservacion del Bosque Chiquitano (FCBC) 2008, Bolivian Road Network Administrator (ABC) 2015.
Protected areas (all categories and grouping all protected areas and indigenous lands)	n/a	National Service of Protected Areas (SERNAP) 2005, Fundación para la Conservacion del Bosque Chiquitano (FCBC) 2011.
Temperature (anomalies corrected for land use change and means estimated based on land surface temperature data for the period 2000 to 2010 from MODIS Terra (MOD11C3)	0.05º	Land Proceses Distributed Active Archive Center (LPDAAC) supported by the National Aeronautics and Space Administration (NASA) and the U.S. Geological Survey (USGS) 2014.
Precipitation (maximum climatological water deficit (MCWD) and MCWD anomalies estimated using monthly rainfall data for the period 2000 to 2010)	0.25º	NASA Tropical Rainfall Measuring Mission (TRMM) 2014.
Hotspots (corresponding to MODIS Aqua and Terra MCD14ML hotspots for the period 2001-2010 (version 5.1) filtered for high confidence >80%)	1 km	NASA Fire Information for Resource Management System (FIRMS) 2014.
