# Supplementary material for: Increased Wildfire Risk Driven by Climate and Development Interactions in the Bolivian Chiquitania, Southern Amazonia
Source: PLoS One. 2016 Sep 15;11(9):e0161323. doi: 10.1371/journal.pone.0161323 (PMC5025183; doi:10.1371/journal.pone.0161323)
Supplement: S2 Table — (PDF) [file pone.0161323.s012.pdf]

Code	Original category	Code	New category†
1	ANMI National	1	National protected area
1	National park	2	Departmental protected area
3	Water body	3	Water body§
5	Intensive cattle ranching	4	Extensive cattle ranching
6	Permanent forest production	5	Intensive cattle ranching and agriculture
7	Forest use and regulated cattle ranching	6	Permanent forest production
6	Forest under protection	7	Forest use and regulated cattle ranching
4	Extensive cattle ranching with forest management	8	Agro-silvopastoral use
8	Silvopastoral use		
4	Extensive cattle ranching		
5	Intensive cattle ranching and agriculture		
4	Extensive cattle ranching with fauna management		
8	Limited forest use		
8	Limited agro-silvopastoral use		
8	Agro-silvopastoral use		
2	AP M Tucabaca Valley, S Chochis, S Santiago		
2	RVS Departmental		
4	Extensive cattle ranching with irrigation potential		

† The original categories of the PLUS in the Chiquitano Model Forest region were clustered into new categories merging the ones that were similar or occupied only very small areas.

§ Water bodies were removed from the dataset for the analysis.
